# Cold-Shock Domain Family Proteins (Csps) Are Involved in Regulation of Virulence, Cellular Aggregation, and Flagella-Based Motility in *Listeria monocytogenes*

**DOI:** 10.3389/fcimb.2017.00453

**Published:** 2017-10-26

**Authors:** Athmanya K. Eshwar, Claudia Guldimann, Anna Oevermann, Taurai Tasara

**Affiliations:** ^1^Institute for Food Safety and Hygiene, Vetsuisse Faculty, University of Zurich, Zurich, Switzerland; ^2^Neuropathology—Division of Neurological Sciences, Vetsuisse Faculty, University of Bern, Bern, Switzerland

**Keywords:** *Listeria monocytogenes*, Csps, virulence, cellular aggregation, flagella, swarming motility

## Abstract

Cold shock-domain family proteins (Csps) are highly conserved nucleic acid binding proteins regulating the expression of various genes including those involved in stress resistance and virulence in bacteria. We show here that Csps are involved in virulence, cell aggregation and flagella-based extracellular motility of *Listeria monocytogenes*. A *L. monocytogenes* mutant deleted in all three *csp* genes (Δ*cspABD*) is attenuated with respect to human macrophage infection as well as virulence in a zebrafish infection model. Moreover, this mutant is incapable of aggregation and fails to express surface flagella or exhibit swarming motility. An evaluation of double *csp* gene deletion mutant (Δ*cspBD*, Δ*cspAD* and Δ*cspAB*) strains that produce single *csp* genes showed that there is redundancy as well as functional differences among the three *L. monocytogenes* Csps in their contributions to virulence, cellular aggregation, flagella production, and swarming motility. Protein and mRNA expression analysis further showed impaired expression of key virulence and motility genes in the *csp* mutants. Our observations at protein and mRNA level suggest Csp-dependent expression regulation of these genes at transcriptional and post-transcriptional levels. In a mutant lacking all *csp* genes (Δ*cspABD*) as well as those possessing single *csp* genes (Δ*cspBD*, Δ*cspAD*, and Δ*cspAB*) we detected reduced levels of proteins or activity as well as transcripts from the *prfA, hly, mpl*, and *plcA* genes suggesting a Csp-dependent transcriptional regulation of these genes. These *csp* mutants also had reduced or completely lacked ActA proteins and cell surface flagella but contained elevated *actA* and *flaA* mRNA levels compared to the parental wild type strain suggesting Csp involvement in post-transcriptional regulation of these genes. Overall, our results suggest that Csps contribute to the expression regulation of virulence and flagella-associated genes thereby promoting host pathogenicity, cell aggregation and flagella-based motility processes in *L. monocytogenes*.

## Introduction

The Gram-positive bacterium *Listeria monocytogenes* is an opportunistic foodborne pathogen that poses a serious public health risk if introduced into the food chain (Allerberger and Wagner, 2010; Anonymous, 2016). The ingestion of contaminated food can lead to listeriosis, a disease associated with severe illnesses, high mortality, abortions, and stillbirths in susceptible or immunocompromised human individuals (Allerberger and Wagner, 2010; Silk et al., 2012). Besides these serious human health risks, listeriosis is also responsible for significant food hygiene challenges and substantial economic losses to the food industry (Kramer et al., 2005; Jami et al., 2015; Melo et al., 2015). *L. monocytogenes* occurs ubiquitously in the environment and can survive in a wide range of environmental conditions. Molecular mechanisms governing resilience in unfavorable conditions, e.g. those associated with food preservation measures or host defenses, as well as the expression of virulence factors that facilitate host cell invasion have been a focus of intense investigation in this bacterium (Dussurget, 2008; Freitag et al., 2009; Soni et al., 2011; Melo et al., 2015).

Upon oral ingestion, various virulence factors mediate the invasion of *L. monocytogenes* into host cells and facilitate spread to neighboring cells (Dussurget, 2008; Freitag et al., 2009). These processes are tightly regulated, mainly by the transcriptional regulator PrfA that gets activated upon host infection (de las Heras et al., 2011). The entry into non-phagocytic host cells depends on InlA and InlB surface proteins, whereas uptake into phagocytic host cells is by phagocytosis (Gaillard et al., 1991; Dramsi et al., 1995). A combination of Listerolysin O (LLO), phospholipases (PlcA and PlcB) and the metalloprotease Mpl facilitate the escape of *L. monocytogenes* from the internalization vacuoles or phagosomes into the host cell cytosol where the bacteria replicate (Portnoy et al., 1988; Camilli et al., 1993; Slepkov et al., 2010). Intracellular motility and cell-to-cell spread is mediated through the surface protein ActA and the internalin protein InlC (Kocks et al., 1992).

Besides these well-defined virulence factors, the expression of flagella and flagella-based motility has also been implicated in virulence functions in various bacteria including *L. monocytogenes* (Josenhans and Suerbaum, 2002; Duan et al., 2013). Flagella provide *L. monocytogenes* with a crucial advantage compared to non-motile bacteria inside (Dons et al., 2004; Bigot et al., 2005; O'Neil and Marquis, 2006) as well as outside the host by enabling the bacteria to actively evade negative stimuli and migrate toward nutrients, to grow at low temperatures (Mattila et al., 2011), or by mediating surface attachment and biofilm formation (Vatanyoopaisarn et al., 2000; Lemon et al., 2007).

Bacterial cold shock-domain protein family proteins (Csps) are small, highly conserved nucleic acid binding proteins that are involved in regulation of various gene expression events (Horn et al., 2007; Keto-Timonen et al., 2016). Occurring in a broad range of bacterial species, these proteins were originally discovered in connection with cold adaptation functions, but they have now been subsequently linked to roles in normal growth as well as regulation of stress adaptation and virulence-associated responses in different bacteria (Horn et al., 2007; Phadtare and Severinov, 2010; Michaux et al., 2012, 2017; Sahukhal and Elasri, 2014; Keto-Timonen et al., 2016; Wang et al., 2016). Although the molecular mechanisms underpinning the Csp-dependent regulation of gene expression are not fully understood, they appear to include nucleic acid binding events allowing modulation of transcription, mRNA stability, translation, DNA replication, and chromosomal condensation processes (Feng et al., 2001; Yamanaka et al., 2001; Phadtare and Severinov, 2010; Batte et al., 2016; Michaux et al., 2017).

*L. monocytogenes* produces three highly conserved Csps named CspA, CspB, and CspD, which similar to other bacteria appear crucial in regulation of stress resistance and virulence related functions in this bacterium (Schmid et al., 2009). Early studies suggested a role for Csps in the adaptation to environmental stresses relevant to the food processing environment since *csp* mutants of this bacterium showed reduced fitness during cold growth, as well as under NaCl and oxidative stress conditions (Wemekamp-Kamphuis et al., 2002; Chan et al., 2007; Schmid et al., 2009; Loepfe et al., 2010). Apart from stress survival functions, Csps also seem crucial in the regulation of virulence functions in *L. monocytogenes* as we previously also discovered that a *cspABD* gene deletion that removes all *csp* genes, does not only reduce the invasion capacity of this bacterium in human Caco-2 and murine macrophage cell lines, but also leads to reduced LLO secretion relative to the parental wild type strain (Loepfe et al., 2010; Schärer et al., 2013). These previous results have led us to hypothesize that, in addition to functions in stress protection, Csps are involved in cell regulatory networks governing the expression of virulence in *L. monocytogenes*.

Our aims in this study were to further assess such roles of Csps in *L. monocytogenes* virulence *in vitro* using a human macrophage cell line as well as *in vivo* using a zebrafish embryo based infection model. Zebrafish embryos are an attractive multicellular model for infection studies, that are easily accessible for microscopy due to their translucent nature, and have been widely used in microbial research with various bacteria including *L. monocytogenes* (Prajsnar et al., 2008; Levraud et al., 2009; Widziolek et al., 2016). Observations from these virulence studies also led us to examine the role of Csps in cellular aggregation as well as in extracellular motility and flagella production of *L. monocytogenes*. A set of *L. monocytogenes csp* mutant strains that either lack all (Δ*cspABD*) or retain only one (Δ*cspBD*, Δ*cspAD*, and Δ*cspAB*) of the three *csp* genes found in this bacterium were used to assess for phenotypes and the expression regulation of selected target genes of the *L. monocytogenes* Csp regulon. Our findings show that besides promoting survival and growth during human macrophage infection and virulence in zebrafish embryos, Csps also facilitate cell aggregation, flagella production, and swarming motility in *L. monocytogenes*. An expression analysis of specific virulence and motility associated genes indicate that Csps are an integral part of the regulatory circuitry that controls expression of virulence and flagella associated genes. Our observations at protein and mRNA levels suggest that Csps probably accomplish their roles by influencing the expression of key virulence and motility associated genes at transcription and translation levels in *L. monocytogenes*.

## Materials and methods

### Ethics statement

Animal research during this study was conducted in accordance with recommendations of approval No. 216/2012 and following the guidelines provided by the Veterinary Office of the Public Health Department of the Canton of Zurich (Switzerland). The number of dead larvae post infection was determined at various time points visually based on the lack of a heartbeat. Experiments were carried out until 72 h post infection (hpi) and at the end of the experiments embryos that were alive were euthanized with an overdose of 4 g L^−1^ buffered tricaine. Usually, with the evaluation of distress and pain by behavioral observations, embryos were euthanized by prolonged immersion in overdose of tricaine solution and were left in the solution for at least 10 min after cessation of opercular movement. Since pain sensitivity has not been developed at these earlier stages (4 dpf−7 dpf), this is not contemplated as a painful technique. The maximum age attained by the embryos throughout investigation was 72 hpf and embryos had not yet reached free feeding stage.

### Bacterial strains and growth conditions

The WT and *csp* deletion mutants *L. monocytogenes* EGDe used in this study are described in Table 1. All *csp* mutants were constructed in-frame as previously described (Schmid et al., 2009). Green fluorescent protein (GFP) expressing derivatives of the strains were generated through site specific PSA-integrase mediated single copy integration of the pPL3-eGFP plasmid (Shen and Higgins, 2005) into the tRNA-Arg locus (Lauer et al., 2002). Sixteen hours secondary stationary phase cultures that were confirmed through optical density measurements and viable cell counting were used in this study. Primary cultures were prepared by inoculating 10 mL BHI (BHI; Oxoid, Hampshire, UK) broth and growing for 16 h at 37°C and 150 rpm. Secondary cultures were subsequently prepared by inoculating 10 ml BHI with these primary cultures (1:1,000) and growing for 16 h at 37°C and 150 rpm. Optical density measurement (OD_600_) and viable cell count based growth curves conducted for each strain showed that all secondary cultures grown in this way and subsequently used for experiments were in the stationary growth phase stage. The pPL3-eGFP integrated strains were similarly grown on BHI agar and broth medium supplemented with erythromycin at 5 μg ml^−1^.

**Table 1:** Bacteria strains and plasmids used in this study.

**Strains and plasmids**	**Description**	**References**
***L. monocytogenes*** **EGDe STRAINS**		
EGDe WT	WT, serotype 1/2a, ATCC BAA-679	Glaser et al., 2001
EGDe Δ*cspABD*	In-frame *cspA, B and D* deletions	Schmid et al., 2009
EGDe Δ*cspBD*	In-frame *cspB* and *D* deletions	Schmid et al., 2009
EGDe Δ*cspAD*	In-frame *cspA* and *D* deletions	Schmid et al., 2009
EGDe Δ*cspAB*	In-frame *cspA* and *B* deletions	Schmid et al., 2009
**GFP LABELED** ***L. monocytogenes***		
EGDe strains		
EGDe WT::pPL3e-GFP	EGDe WT with pPL3e-gfp integration into the tRNA^Arg^ locus	This study
EGDe Δ*cspABD*::pPL3e-GFP	EGDe Δ*cspABD* with pPL3e-*gfp* integration into the tRNA^Arg^ locus	This study
EGDe Δ*cspBD*:: pPL3e-GFP	EGDe Δ*cspBD* with pPL3e-*gfp* integration into the tRNA^Arg^ locus	This study
EGDe Δ*cspAD*:: pPL3e-GFP	EGDe Δ*cspAD* with pPL3e-*gfp* integration into the tRNA^Arg^ locus	This study
EGDe Δ*cspAB*:: pPL3e-GFP	EGDe Δ*cspAD* with pPL3e-*gfp* integration into the tRNA^Arg^ locus	This study
**PLASMIDS**		
pPL3e-*gfp*	Integrative plasmid vector pPL3e—*gfp* for the constitutive expression of green fluorescence protein (GFP)	Shen and Higgins, 2005

### THP1 cell culture

THP-1 cells (ATCC TIB-202) were maintained in T75 tissue culture flasks (TPP- Techno plastic products, Switzerland) and grown to confluence in RPMI 1640 medium (RPMI; Sigma Aldrich, Germany) containing 0.3 g l^−1^ L-glutamine, 2 g l^−1^ sodium bicarbonate supplemented with 10 mM HEPES (Sigma Aldrich, Buchs, Switzerland), 1 mM sodium pyruvate (Sigma Aldrich, Buchs, Switzerland), 4.5 g l^−1^ glucose and 10 % fetal bovine heat-treated (56°C, 30 min) serum (Sigma Aldrich, Buchs, Switzerland Germany) and incubated at 37°C with 5% CO_2_.

### Gentamicin protection assays

THP-1 cells were seeded at a density of 10^5^ cells per well in 24-well tissue plates and incubated for at least 24 h (37°C and 5% CO_2_) in RPMI 1640 containing 0.1 μg ml^−1^ of phorbol 12-myristate 13-acetate (PMA; Sigma Aldrich, Buchs, Switzerland) to induce stable differentiation of the THP-1 monocytes into macrophages. The PMA containing medium was removed and the cells washed once using 500 μl RPMI 1640. THP-1 macrophages were infected at ratio of 10 bacteria organisms per cell using *Listeria* that had been cultivated as described above and diluted in RPMI 1640. After 45 min (37°C and 5% CO_2_) of incubation the macrophages were washed twice using DPBS (500 μl) before incubation for 45 min in RPMI 1640 (500 μl) containing 50 μg ml^−1^ gentamicin to kill extracellular bacteria. The macrophages were washed once using 0.5 ml of antibiotic free RPMI 1640 after this step and then incubated in 0.5 ml RPMI 1640 that contained 10 μg ml^−1^ gentamicin. Infections immediately stopped at this point represented time point *t*_0_ and the initial macrophage internalized inoculum. For the rest of the samples incubation was continued and infections were stopped after 6 (*t*_6_) and 24 (*t*_24_) h of macrophage infection. To stop infections the macrophages were washed twice using 0.5 ml DPBS and then lysed using 0.5 ml of DPBS plus 0.5% Triton X-100. The cell lysates were 10-fold serially diluted, plated out on plate count agar plates (PC agar, Sigma) and incubated for 24 h at 37°C before enumerating the number of viable intracellular bacteria. Intracellular amounts of bacteria (IBC) CFUs (colony forming units) present at 6 (*t*_6_) and 24 (*t*_24_) h of THP-1 macrophage infection were determined and expressed as a percentage relative to the initial intracellular inoculum found in macrophages at time point zero (*t*_0_) as follows: (IBC*t*_*n*_ – IBC*t*_0_/IBC*t*_0_) × 100, where IBCt_0_ and IBCt_n_ are the intracellular bacterial CFUs determined at time points zero (*t*_0_) and *n* (*t*_6_ and *t*_24_), respectively.

### Confocal laser scanning microscopy

GFP expressing EGDe WT and *csp* mutant strains (Table 1) were used in macrophage infection assays with subsequent microscopic analysis. In each well of the 24 well plate, a sterile glass coverslip (13 mm in diameter, Menzel-Gläser) was placed and THP-1 cells were seeded on the coverslips. Cell culture, PMA activation and infection assays were carried out as mentioned above. At each indicated time point (*t*_0_, *t*_6_, and *t*_24_), THP-1 macrophages adhered to coverslips were gently washed twice with DPBS and fixed with 4% paraformaldehyde (PFA, Sigma) at 4°C for 15 min. After fixation, the PFA was removed, the macrophages were gently washed twice with DPBS to remove residual PFA, and the samples were stained for 1 h at room temperature with Hoechst Dye (Life Technologies, Zug, Switzerland) and Concanavalin A Alexa Fluor® 594 Conjugate (Life Technologies). After staining, the macrophages were washed 3–5 times with DPBS, mounted on glass slide using Fluoromount (Sigma) mounting medium, air dried in the dark and imaged using a Leica TCS SP5 Confocal microscope (63x or 40x oil-immersion objective, excitation at 405 nm for Hoechst, 488 nm for GFP and 594 nm for Concanavalin A).

### Zebrafish lines and husbandry

Zebrafish (*Danio rerio*) strains used in this study were *wik* lines. Adult fish were kept at a 14/10 h light/dark cycle at a pH of 7.5 and at 27°C. Eggs were obtained from natural spawning between adult fish which were set up pairwise in individual breeding tanks. Embryos were raised at 28°C in petri dishes containing E3 medium (5 mM NaCl, 0.17 mM KCl, 0.33 mM CaCl_2_, and 0.33 mM MgSO_4_) supplemented with 0.3 μg/ml of methylene blue. From 24 h post fertilization (hpf), 0.003% 1-phenyl-2-thiourea (PTU; Sigma Aldrich, Buchs, Switzerland) was added to prevent melanin synthesis. Staging of the embryos was performed according to Kimmel et al. (1995). All zebrafish experiments were conducted with the approval (no. 216/2012) from the Veterinary Office, Public Health Department, Canton of Zurich (Switzerland).

### Microinjection experiments

Injections were performed using borosilicate glass microcapillary injection needles (Science Products, 1210332, 1 mm OD × 0.78 mm ID) and a PV830 Pneumatic PicoPump (World Precision Instruments). Bacteria for microinjection experiments were harvested from stationary phase BHI bacteria cultures by centrifugation at 5,000 × g for 10 min and washed in 10 ml Dulbecco's Phosphate-Buffered Saline (DPBS) before the cells were resuspended in DPBS and appropriate dilutions prepared. Two-day post fertilization (dpf) embryos were manually dechorionated and anesthetized with 200 mg/l buffered tricaine (Sigma Aldrich, Buchs, Switzerland, MS-222) prior to injections. Subsequently, the embryos were aligned on an agar plate and injected with 100 CFU (range: 90–136 CFU) in 1–2 nl volume of a bacterial suspension in DPBS into the yolk sac or blood circulation via the caudal vein close to the urogenital opening. Prior to injections the volume of the injection suspension was adjusted by injecting a droplet into mineral oil and measuring its diameter over a micrometer scale bar. The number of CFU injected was determined by direct microinjection of a DPBS droplet on agar plates and confirmed by disintegrating five embryos individually immediately after microinjection (0 hpi) and plating the lysates on LB agar. Post injection the infected embryos were allowed to recover in a petri dish in fresh E3 medium for 15 min. To follow the infection and mortality kinetics, embryos were transferred into 24-well plates (one embryo per well) in 1 ml E3 medium per well, incubated at 28°C and observed for signs of disease and survival under a stereomicroscope twice a day. For survival assays after infection, the number of dead larvae was determined visually based on the absence of a heartbeat. At each time point, five embryos or larvae were collected, euthanized, and individually treated for bacterial enumeration. For subsequent microscopic analyses larvae were euthanized with an overdose of 4 g/l buffered tricaine and transferred into respective buffers and fixatives.

### Bacterial enumeration by plate counting

The larvae were transferred to a 1.5 ml Eppendorf tube containing 1 ml DPBS supplemented with 1% Triton X-100 and disintegrated by repeated pipetting and vortexing for 3 min. Subsequently, 100 μl of this mixture was plated onto BHI selective plates (i.e., erythromycin 5 μg l^−1^ for strains harboring pPL3::GFP) and the plates were incubated up to 48 h at 37°C.

### RNA extraction and RT-qPCR

Reverse transcription quantitative-PCR (RT-qPCR) was used to determine transcript levels of selected genes in EGDe WT and the *csp* mutant strains. Bacteria grown to stationary phase in BHI as described above or recovered from THP-1 macrophages that were infected for 6 h with *Listeria*, and then washed and lysed as described above were used. *L. monocytogenes* cells (10^9^ CFUs) in 1 ml of stationary phase BHI broth cultures prepared as described above were harvested in RNA protect Bacteria reagent (Qiagen AG, Hombrechtikon, Switzerland) and resuspended using 0.5 ml of the RNeasy Plus Mini Kit (Qiagen AG, Hombrechtikon, Switzerland) lysis buffer. Bacteria recovered from macrophage lysates by centrifugation were similarly harvested in RNA-protect Bacteria Reagent and subsequently re-suspended in the lysis buffer. *L. monocytogenes* cells resuspended in the lysis buffer were transferred into beads in MagNA lyser tubes and mechanically disrupted using the MagNA Lyser Instrument (Roche Molecular Diagnostics, Rotkreuz, Switzerland). RNA was subsequently isolated from the lysates following the RNeasy Plus Mini Kit protocol. Purified RNA was quantified using a Nanodrop (Thermo Scientific, USA) and its quality was verified using a BioAnalyzer (Agilent technologies, USA). cDNA was synthesized from 100 ng of RNA samples with RNA integrity numbers (RINs) of 7 or above. The Quantitect Reverse Transcription Kit (Qiagen AG, Hombrechtikon, Switzerland) was used. Residual DNA contamination of RNA samples was ruled out through the inclusion of no RT controls in the analysis. 2.5 ng of the cDNA were used as templates for the RT-qPCR. Primers listed in Table 2 and the SYBR green I kit (Roche Molecular Diagnostics, Penzbrug, Germany) were used for the RT-qPCR in a LC480 instrument (Roche Molecular Diagnostics, Rotkreuz, Switzerland). Relative cDNA quantification was performed using the Light Cycler 480 Relative Quantification Software (Roche Molecular Diagnostics). Amounts of the transcript amounts were normalized to 16S rRNA as reference gene. Using a delta-delta CT approach, transcript levels of each gene were expressed relative to the values of a calibrator mRNA sample, which was derived from stationary phase EGDe WT culture grown in BHI broth.

**Table 2:** Primers used in this study.

**Gene target**	**Primer sequence (5′–3′)**	**Protein**
*16S rDNA*	Fwd—CTTCCGCAATGGACGAAAGT	Small ribosomal RNA subunit
	Rev—CTCATCGTTTACGGCGTG	
*mpl*	Fwd—TCAGGTGCGCTAAACG	Metalloprotease
	Rev—GTCGCCTTCCTCTGTG	
*actA*	Fwd—GCACCGGCTCTGATAAG	Actin assembly-inducing protein (ActA)
	Rev—GGTAGGCTCGGCATATT	
*plcA*	Fwd—TCGGGGAAGTCCATGA	Phosphatidylinositol phospholipase C (PI-PLC)
	Rev—GGCGCACCTAACCAAG	
*hly*	Fwd—ACCTCGGAGACTTACG	Listeriolysin O (LLO)
	Rev—TCCTCCAGAGTGATCG	
*prfA*	Fwd—TGGTATCACAAAGCTCACG	Positive regulatory factor A (PrfA)
	Rev—TGGTATCACAAAGCTCACG	
*flaA*	Fwd—CAGCGGATTCAGCTCT	Flagellin protein FlaA
	Rev—CGGATAATGCACTATAACCAG	

### Immunoblotting

Virulence protein expression was assessed in both extracellular and intracellular grown *L. monocytogenes*. To assess protein expression in extracellular bacteria, *L. monocytogenes* were cultured to stationary phase in BHI (30 ml; 16 h at 37°C and 150 rpm) as described above, standardized to OD600 of 1.0 by dilution in BHI broth and the cells were collected by centrifugation (10,000 g for 2 min) at 4°C. To assess intracellular virulence protein expression THP-1 macrophages infected with Listeria for 6 h. Post infection the THP-1 macrophages were washed and lysed to release intracellular bacteria as described above. *L. monocytogenes* were recovered from the THP-1 macrophage lysates by centrifugation (10,000 g for 10 min) at 4°C.

To extract proteins, *L. monocytogenes* pellets were resuspended in RIPA lysis buffer (1 ml) containing a protease inhibitor cocktail (Cell Biolabs Inc, San Diego, USA) and mechanically disrupted (2 × 6,500 rpm for 1 min) in the MaGNA lyser instrument (Roche Molecular Diagnostics). The resulting *L. monocytogenes* lysates were cleared by centrifugation (10,000 g for 10 min at 4°C) and protein concentration in the supernatants was determined. Equal amounts of proteins (20 μg) from each sample were separated by sodium dodecyl sulfate–polyacrylamide gel electrophoresis (SDS-PAGE; 4–20%) and transferred onto PVDF membranes. The membranes were probed with primary rabbit polyclonal antibodies raised against PrfA (1:5,000), Mpl (1:5,000) and LLO (Diatheva, Fano PU, Italy; 1:1,000), as well as primary mouse monoclonal antibodies directed against ActA (Abanova, Taipei, Taiwan, 1:1,000) and P60 (Adipogen, San Diego, USA; 1:2,000). A HRP-conjugated anti-rabbit IgG (Sigma-Aldrich, Buchs, Switzerland; 1:2,000) secondary antibody was subsequently used to probe all blots except the ActA and P60 blots, which were probed using an HRP-conjugated anti-mouse IgG (Sigma-Aldrich, Buchs, Switzerland; 1:100,000) secondary antibody. The primary and secondary antibodies were diluted in 1% skimmed milk solution prepared in tris buffered saline with tween 20. The densitometric quantification of the proteins was carried out using the ImageJ software (National Institute of Health, USA. The intensities of PrfA, Mpl, LLO, and ActA bands obtained by chemiluminescence were normalized using the intensity of P60 from the corresponding sample.

### Analysis of phosphatidylinositol-specific phospholipase C (PI-PLC) activity, aggregation, and swarming motility

To compare PI-PLC activities, the colonies of *L. monocytogenes* strains were streaked on ALOA (Agar Listeria according to Ottaviani and Agosti) plates, incubated at 37°C and visually examined after 48 h for the zone of opacity. Aggregation of Listeria strains was compared as previously described (46). In short, 16 h stationary phase BHI cultures of WT and *csp* mutant *L. monocytogenes* strains were adjusted to OD_600_ of 3.5 in BHI. The adjusted cultures were statically incubated at 37°C for 24 h and monitored through visual and microscopic examination for aggregation. Aggregation was indicated by decreasing cultural supernatant optical density and the development of bacterial sediments. To determine the optical density, aliquots were collected ~1 cm from the top of each sample at defined time intervals to measure OD_600_.

Swarming motility was compared by spotting 16-h stationary phase BHI cultures from each strain (5 μl) onto the surfaces of 0.25% BHI agar plates and incubating 48 h at 25°C. For electron microscopic examination bacteria colonies grown overnight at 25°C on a 0.25% BHI agar plate were picked up using an inoculation loop and transferred to an Eppendorf tube containing 100 μl of 2.5% glutaraldehyde and incubated for at least 30 min at room temperature to fix. Further, 1:1 suspensions were prepared using 0.1 M sodium phosphate buffer and bacteria-glutaraldehyde mix. These suspensions were then transferred onto 150 mesh copper grids with Formvar carbon film (Electron Microscopy Sciences, USA) and incubated at room temperature for 2 min for adhesion. Following adhesion, bacterial flagella were negatively stained with uranyl acetate for 10 s and with 1:1 suspension of uranyl acetate and water for 20 s, and observed in an electron microscope.

### Statistical analysis

Statistical analyses were carried out using JMP software (Version 12.1.0, SAS Institute Inc., NC, USA). All experiments presented were performed independently at least three times. One way ANOVA with *post-hoc* Tukey HSD tests were used to assess statistical significance of differences relative to the WT as well as between the different *csp* mutant strains in macrophage and zebra infection as well as RT-qPCR assays. Kaplan Meier survival analysis and statistics for experiments with zebrafish were done with GraphPad Prism 7 software (GraphPad Software, United States). *p* < 0.05 were considered to be statistically significant.

## Results

### Csp loss impairs survival and growth of *L. monocytogenes* in human macrophages

**Figure 1:**
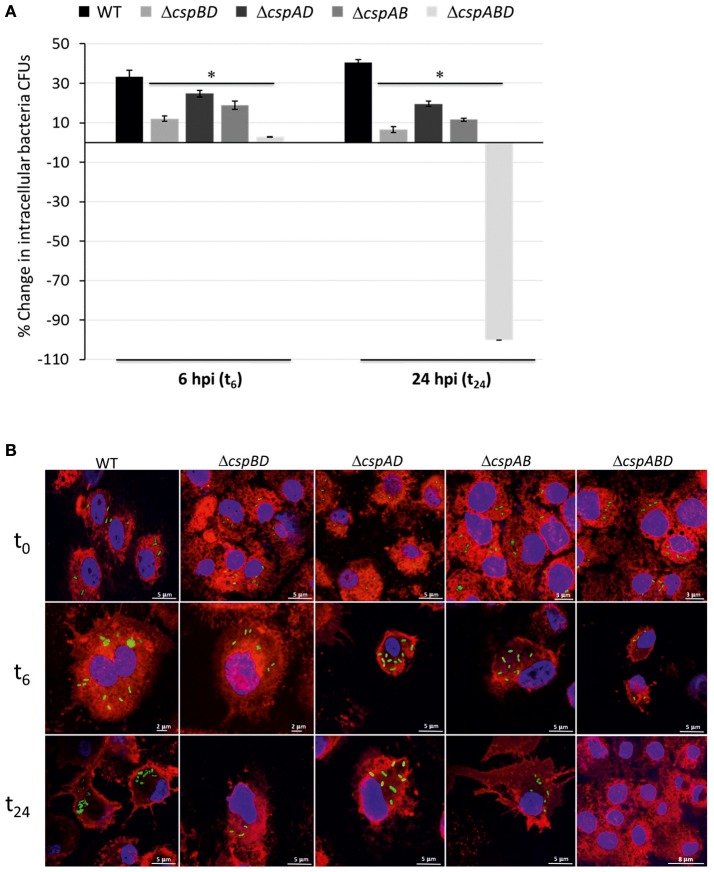
Csp loss impairs survival and growth of *L. monocytogenes* in human macrophages. **(A)** Bar charts depicting percentage change in intracellular bacterial CFUs for the WT and *csp* mutants of *L. monocytogenes* EGDe at 6 (*t*_6_) and 24 (*t*_24_) h post infection (hpi) of human derived THP-1 macrophages. The percentage changes (CFUs) are expressed relative to the intracellular bacteria CFUs determined at time point 0 (*t*_0_). Data showing the means and standard deviations derived from three independent biological experiments are presented. ^*^p < 0.05 based on one-way ANOVA and Tukey *post-hoc* test comparison relative to the WT strain as well as between the different *csp* mutants. **(B)** Representative images from the fluorescent microscopic analysis based monitoring of the intracellular fates of GFP expressing WT and *csp* mutants of *L. monocytogenes* EGDe strain during THP-1 macrophage infections at 0(*t*_0_), 6(*t*_6_), and 24(*t*_24_) hpi.

Since the survival and multiplication of *L. monocytogenes* within target host cells is crucial step for successful host infections, we initially examined the functional contribution of Csps in this bacterium during human macrophage infection in cell culture. The survival and growth of WT and *csp* gene deletion mutant strains of *L. monocytogenes* EGDe during the infection of human derived THP-1 macrophages was compared. Based on comparison of viable bacteria CFUs that were recovered inside macrophages at infection time point zero (*t*_0_) we determined that there are similar levels of bacteria that were initially internalized into THP-1 macrophages for the WT strain as well as all the *csp* mutants (Figure S1). Subsequent monitoring of changes in intracellular bacterial CFUs conducted after 6 (*t*_6_) and 24 (*t*_24_) h of macrophage infection however showed that the complete deletion of all the three *csp* genes (Δ*cspABD*), as well as the presence of only single *csp* genes (Δ*cspBD*, Δ*cspAD*, and Δ*cspAB*) is associated with impaired survival and growth of *L. monocytogenes* in THP-1 macrophages (Figure 1A). In samples examined at 6 h (*t*_6_) post infection, the WT strain bacteria levels inside macrophages had increased by an average of 33.3% when compared to the inoculum found inside macrophages at 0 (*t*_0_) h of infection. But a Δ*cspABD* mutant deleted in all the three *csp* genes only displayed limited growth with its levels inside macrophages having increased by an average of only 2.8% at this time point. At 24 h (*t*_24_) post infection, the WT strain levels in macrophages showed further increase (40.5% increase relative to the t_0_ levels), whereas the Δ*cspABD* mutant could not be detected in THP-1 macrophage infected for 24 h. This suggests that both long-term growth and survival of *L. monocytogenes* inside human macrophages are significantly impaired without Csps (Figure 1A).

A caveat in assessing the functional role of individual *csp* genes in *L. monocytogenes* using single gene deletion mutant backgrounds is that some phenotypes might be masked due to functional redundancy that exist between the three *csp* genes found in this bacterium. To overcome this caveat and allow us to confirm as well as assess the level of phenotypic contributions of the individual *csp* genes during THP-1 macrophage infections, three mutant strains, which are deleted in two leaving only one of the three *csp* genes intact were analyzed. To this end the Δ*cspBD*, Δ*cspAD*, and Δ*cspAB* mutant strains expressing CspA, CspB, and CspD, respectively, were compared with respect to growth and survival in human macrophages. We found that the survival and growth phenotypes achieved by the three single Csp producing strains in THP-1 macrophages although variable were all significantly above those observed for Δ*cspABD* mutant, which lacks all the three *csp* genes (Figure 1A). These observations thus did not only confirm that all three *csp* genes were functionally relevant but that they also contribute to varying extents toward *L. monocytogenes* survival and growth in human macrophages. We found that none of the three Csps when produced alone is capable of restoring the WT phenotypic levels without the other two *csp* genes (Figure 1A). Overall there was a clear hierarchical trend of WT > Δ*cspAD* (CspB) > Δ*cspAB* (CspD) > Δ*cspBD* (CspA) > Δ*cspABD* (Figure 1A; *P* < 0.05) observed with respect to the intracellular bacteria CFU levels reached inside the THP-1 macrophages at 6 and 24 h post infection (Figure 1A).

We similarly also used GFP expressing bacteria and fluorescent microscopy to qualitatively follow the intracellular fates of WT and *csp* mutant strains during THP-1 macrophage infections. This approach despite being qualitative revealed that intracellular multiplication and survival of the Δ*cspABD* mutant without Csps was impaired compared to the WT strain during human macrophage infection (Figure 1B). Samples collected immediately post macrophage infection at the t_0_ time point showed no microscopically visible differences with respect to macrophage internalized bacteria observed inside infected THP-1 macrophages between the WT strain and the different *csp* mutants (Figure 1B). Such observations were therefore consistent with viable bacterial counts results that had quantitatively shown no significant differences in the initial macrophage entry between WT and the *csp* mutant strains (Figure S1). Significant differences were however observed between the WT and the *csp* mutant strains upon examination of samples collected 6 (*t*_6_) and 24 (*t*_24_) h after infection (Figure 1B). At both these time points samples infected with the WT strain displayed relatively higher amounts of bacteria inside macrophages compared to their *t*_0_ samples as well as to samples of macrophages that were infected with different *csp* mutants. The WT bacteria found inside the macrophages at these points occurred in large clumps consistent with an ongoing multiplication inside the THP-1 macrophages. In contrast, there were relatively fewer and visibly smaller intracellular bacteria clumps observed for the Δ*cspABD* and the three single Csp strains at 6 h (*t*_6_) post infection compared to WT. This was indicative of slower intracellular replication of these mutants compared to WT inside the human macrophages. Moreover, consistent with the quantitative viable bacterial count results there were no Δ*cspABD* detected inside THP-1 macrophages infected for 24 (*t*_24_) h with this mutant (Figure 1B). Although all three single Csp strains could be detected and also displayed microscopic evidence of ongoing intracellular multiplication at 6 (*t*_6_) as well as 24 (*t*_24_) h post macrophage infection, the levels of intracellular replicating bacteria observed inside the macrophages for all were visibly lower than the WT strain but more than those observed for the Δ*cspABD* mutant.

Overall our analysis of the different *csp* mutants using this *in vitro* cell culture based virulence model thus indicates that all three Csps are functionally relevant but to varying degrees in promoting *L. monocytogenes* survival and growth during human macrophage infection. Moreover, the fact that none of the three Csps when expressed alone can restore these phenotypes to WT levels indicates that the functional activities of all the three *csp* genes is necessary for maximal survival and growth of this bacterium during human macrophage infection.

### Csp loss attenuates virulence of *L. monocytogenes* in zebra fish embryos

**Figure 2:**
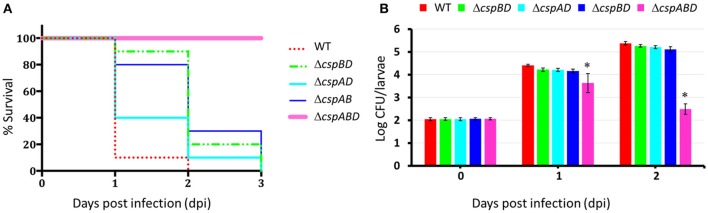
Csp loss attenuates virulence and reduces bacterial loads of *L. monocytogenes* in zebrafish embryos. **(A)** Survival curves were generated for zebrafish embryos (*n* = 5 per *L. monocytogenes* strain and time-point) infected (100 CFU per embryo) with WT and *csp* mutants of *L. monocytogenes* EGDe and monitored over 3 days. Zebrafish embryos rapidly succumbed to the WT strain, while those infected with the *csp* mutants died later or were not killed at all. Survival curves and trends were significantly different (log-rank test *p* < 0.05) between WT and different *csp* mutant strains. **(B)** Bacterial load quantifications were conducted at 1 and 2 days post infection in zebrafish embryos (*n* = 30) infected with WT and *csp* mutant strains. Data showing the mean and standard deviations of three independent biological experiments are presented. ^*^p < 0.05 based on one-way ANOVA and Tukey *post-hoc* test comparison relative to the WT strain.

The impaired survival and growth observed for the different *csp* mutants in human macrophages prompted us to further examine the functional relevance of Csps to *L. monocytogenes* virulence using a zebrafish embryo based multicellular *in vivo* infection model. Zebrafish embryos were infected with WT and *csp* mutants of *L. monocytogenes* EGDe strains by injecting into the blood stream and the capability of the different strains to induce mortality of the infected zebra fish embryos was monitored over a 3 day (72 h) infection period. Survival curves generated showed that while all WT strain infected zebrafish embryos succumbed to the infection within 2 days of infection none of those infected with Δ*cspABD* infected embryos had died even after 3 days post injection (dpi) (Figure 2A). All embryos infected with the three strains harboring single *csp* genes also eventually succumbed but 100% mortality level in all the three strains was delayed occurring a day later than WT at 3 dpi. A comparison of the level of mortality associated with all strains at 1 and 2 dpi showed a hierarchical trend of WT > Δ*cspAD* (*cspB*) > Δ*cspAB* (*cspD*) > Δ*cspBD* (*cspA*) > Δ*cspABD*; *P* < 0.05), which was similar to the survival and growth efficiency trends observed using the THP-1 human macrophage cell infection model (Figure 2A). To additionally assess for differences observed in virulence we monitored for trends in the bacterial loads associated with the WT and *csp* mutant strains during the course of the zebra fish embryo infections. Bacterial CFU levels within infected zebrafish embryos at 0, 1, and 2 dpi were determined. At 0 dpi similar levels of bacterial loads were determined in embryos infected with the WT strain and all the *csp* mutants. In 1 and 2 dpi samples we detected similar progressive increase in the bacterial loads relative to the initial bacterial loads found at 0 dpi (Figure 2B). More importantly there were no significant differences in overall bacterial loads achieved between the WT strain and the three strains possessing single *csp* genes. In contrast, the bacterial loads in Δ*cspABD* mutant infected embryos also increased on 1 dpi relative to 0 dpi, but the bacterial load increases were lower compared to those observed for WT and single Csp strain infected embryos. Furthermore, in contrast to the bacterial loads of the other strains that increased, the Δ*cspABD* bacterial loads showed a significant reduction between 1 and 2 dpi, indicating that the long-term survival of the Δ*cspABD* mutant was also compromised within infected zebrafish embryos. Overall the Δ*cspABD* bacterial loads achieved at 1 and 2 dpi were significantly lower than those of the WT and single Csp strains. Overall our results thus indicated that the three Csps while all functionally relevant also showed variable capacities in enabling full *L. monocytogenes* virulence expression using this *in vivo* multicellular infection model based on zebrafish embryos.

### Csp loss reduces expression of key virulence proteins in *L. monocytogenes*

**Figure 3:**
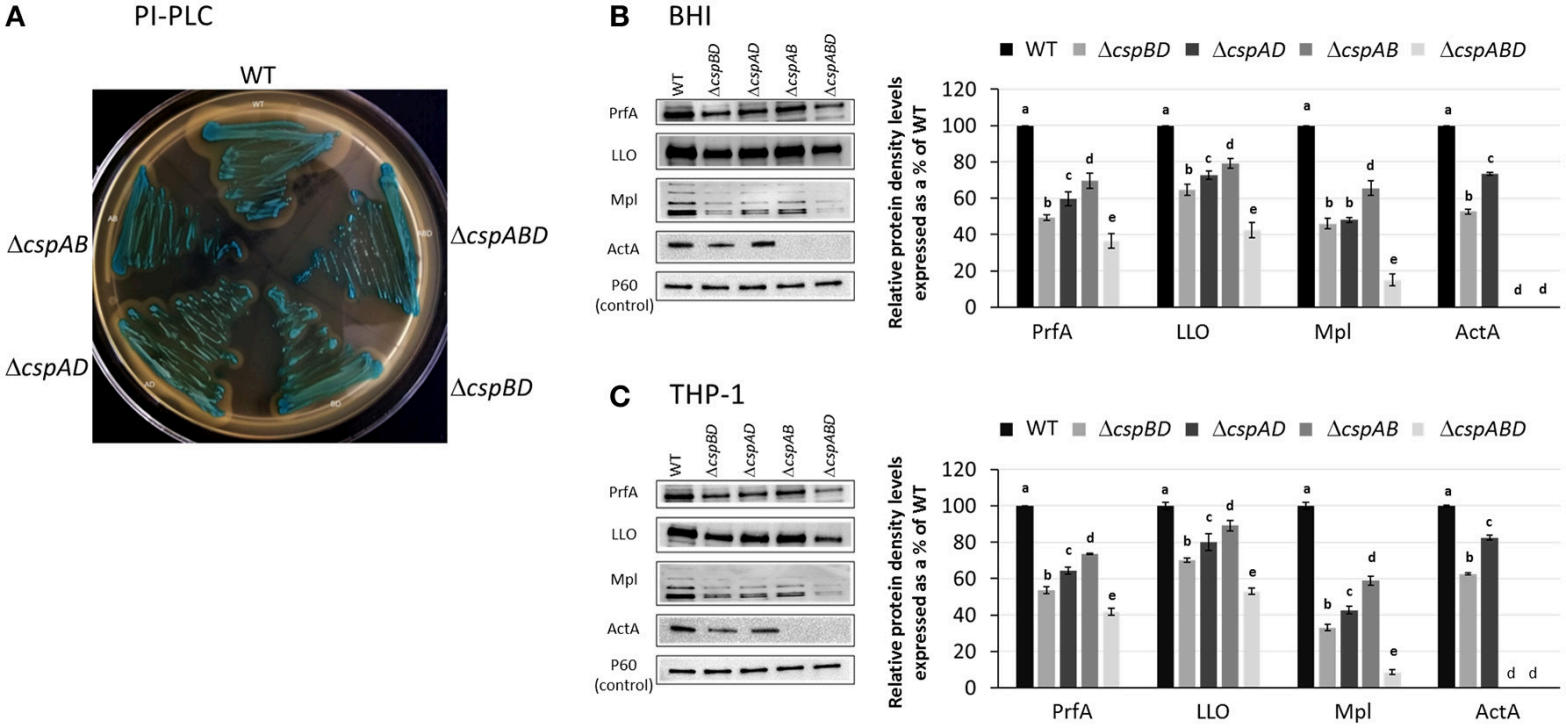
Impact of Csp on the expression of virulence proteins. **(A)** Representative image from the qualitative assessment of PI-PLC activity (turbid zone around streaked bacteria) WT and *csp* mutants of *L. monocytogenes* EGDe grown on ALOA plates. **(B,C)** Representative Western blot images and densitometry based quantification of the PrfA, LLO, Mpl, ActA proteins in BHI cultured (16 h at 37°C and 150 rpm) and THP-1 macrophage grown (6 hpi) WT and *csp* mutants of *L. monocytogenes* EGDe. Virulence proteins densities were normalized to P60 levels and expressed as a percentage relative to WT strain levels. Data showing the means and standard deviations of three independent biological experiments are presented. Bars denoting relative protein abundance levels that share a letter are not significantly different, whereas those marked with different letters are significantly different compared to each other (*p* < 0.05 based on one-way ANOVA and Tukey *post-hoc* test pairwise comparison of all the strains).

In seeking for a possible explanation of the virulence impairment observed in the *csp* mutants we next considered the impact Csp loss on the expression of some key virulence proteins in *L. monocytogenes*. In our previous studies we could show that a strong reduction of LLO protein expression and hemolytic activity was imposed by deletion of *csp* genes in *L. monocytogenes* (Schärer et al., 2013). To examine the impacts of Csp loss on the expression of the other virulence proteins we combined Western blot analysis and densitometry based protein band quantification and compared PrfA, LLO, Mpl, and ActA production between the different *csp* mutants and the parental WT strain (Figure 3, Figures S2–S4). BHI cultured and THP-1 macrophage grown (6 h post macrophage infection) bacteria were used for this analysis. To provide a control and reference protein for the analysis we also determined and quantified the amounts of the secreted P60 protein. Despite all strains showing similar P60 levels, significant differences in PrfA, LLO, Mpl, and ActA levels were detected among the different *csp* mutants as well as in comparison to the WT strain. A Δ*cspABD* mutant lacking all three *csp* genes in particular produced significantly lower amounts of all the examined virulence proteins compared to the WT strain and all three single Csp strains (Figures 3B,C). The three single Csp producing strains in turn all displayed lower levels of these virulence proteins relative to the WT strain. Comparing the single Csp producing strains to each other, we found that PrfA and LLO levels were highest in Δ*cspAB* (*cspD*) followed by Δ*cspAD* (*cspB*) strains, whereas the Δ*cspBD* (*cspA*) consistently showed lowest amounts for these proteins. On the other hand, Mpl levels were similar (BHI cultured bacteria) to slightly different (THP-1 grown bacteria) between the Δ*cspBD* (*cspA*) and Δ*cspAD* (*cspB*) strains, but the levels in these two strains were significantly below those of the Δ*cspAB* (*cspD*) strain. No ActA proteins were detected in the Δ*cspABD* and Δ*cspAB* (*cspD*) strains, whereas ActA amounts found in Δ*cspBD* (*cspA*) and Δ*cspAD* (*cspB*) strains were below WT levels. Among the single Csp producing strains the Δ*cspAD* (*cspB*) strain showed significantly higher ActA levels than the Δ*cspBD* (*cspA*) strain (Figures 3B,C).

**Figure 4:**
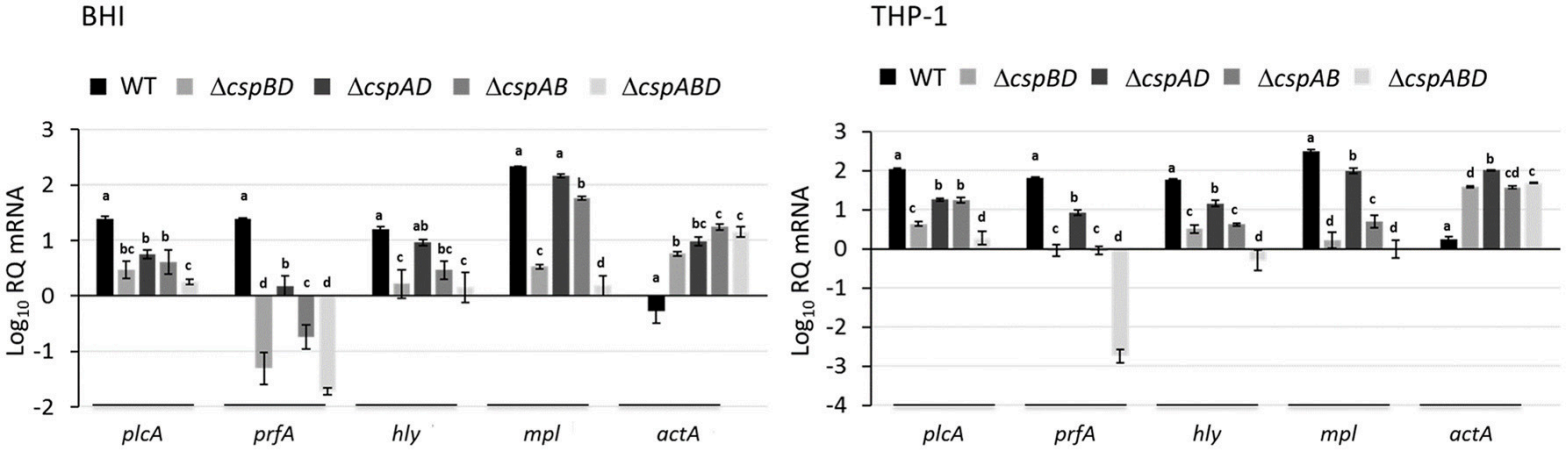
Impact of Csp loss on virulence gene mRNA levels. Quantification of *plcA, prfA, hly, mpl*, and *actA* mRNAs using qRT-PCR in *L. monocytogenes* EGDe WT and *csp* mutant strains that were cultured in BH broth (16 h at 37°C and 150 rpm) and grown in THP-1 macrophage (6 hpi). Relative quantities (RQ) of *plcA, prfA, hly*, and *actA* mRNA levels were normalized to 16S rRNA and expressed relative to those in an EGDe WT strain based mRNA calibrator sample. Data showing the means and standard deviations of three independent biological experiments are presented. Bars denoting relative mRNA abundance levels that share a letter are not significantly different, whereas those marked with different letters are significantly different compared to each other (*P* < 0.05 based on one-way ANOVA and Tukey *post-hoc* test pairwise comparison of all the strains).

The combined results from qualitative PlcA (PI-PLC) activity analysis and Western blot quantification of PrfA, LLO, Mpl, and ActA proteins indicated that the loss *csp* genes significantly reduces virulence protein production. In order to access if reduced virulence protein production is also reflected at transcript level within the different *csp* mutants we used RT-qPCR and compared the *prfA, hly* (LLO), *mpl*, and *actA* mRNA levels between the WT and the *csp* mutants. In RNA isolated from both BHI and THP-1 macrophage grown bacteria, we found that consistent with low PrfA, LLO and Mpl levels observed in Δ*cspABD*, the *prfA, hly*, and *mpl* mRNA levels in this mutant were also significantly lower compared to the WT strain (Figure 4). A comparison with the single Csp strains also showed that the *prfA, hly*, and *mpl* mRNA levels in Δ*cspABD* are generally also lower compared to these strains although in some instances the differences were not statistically significant (Figure 4). The *prfA, hly*, and *mpl* mRNA levels in the single Csp producing strains on the other hand were for most cases also significantly lower than the WT strain, which is consistent with their reduced PrfA, LLO, and Mpl protein levels compared to the WT strain. An exception to such trends was however observed in case of *hly* and *mpl* mRNA levels, which were not significantly different compared to the WT strain in RNA derived from BHI cultured bacteria of the *cspB* harboring Δ*cspAD* mutant (Figure 4). Although containing lower PrfA, LLO, and Mpl protein amounts compared to the *cspD* harboring Δ*cspAB* mutant, the *cspB* harboring Δ*cspAD* mutant however contained higher *prfA, hly*, and *mpl* mRNA amounts (Figures 3, 4). More surprising was the fact that all the *csp* mutants despite presence of lower (Δ*cspBD* and Δ*cspAD*) or no (Δ*cspABD* and Δ*cspAB*) ActA proteins contained significantly more *actA* mRNA compared to the WT strain (Figure 4). As such the reduced amounts and absence of ActA in the *csp* mutants was not associated with a reduction or lack of *actA* mRNA in these mutants (Figures 3, 4). Overall our observations thus showed that the complete removal or the production of only single Csps leads to either a reduction of proteins as well as their corresponding transcripts (PrfA, LLO, and Mpl) or reduced protein levels while the encoding transcripts are present at elevated (ActA) levels.

### Csp loss abrogates cellular aggregation in *L. monocytogenes*

**Figure 5:**
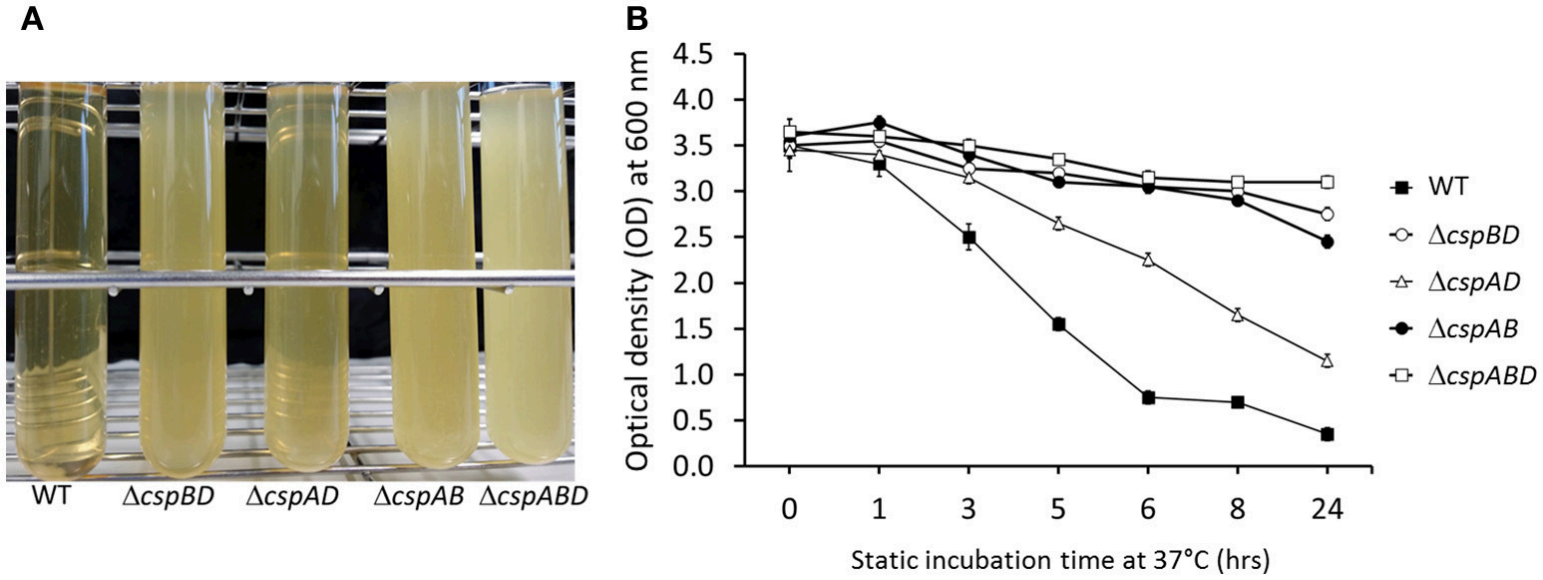
Aggregation analysis in stationary phase BHI cultures of *L. monocytogenes* EGDe WT and *csp* mutant strains incubated for 24 h at 37°C without shaking. **(A)** Bacterial sedimentation observed at 24 h and **(B)** time resolved aggregation kinetics based on changes in the culture supernatant OD_600_ during 24 h of static incubation.

Besides intracellular motility roles, ActA has also been described as one of the major cell aggregation determinants in *L. monocytogenes*. Our observations of reduced amounts and complete lack of ActA amongst the *csp* mutants led us to hypothesize that cell aggregation in *L. monocytogenes* might have also been indirectly compromised due to Csp deficiency. To examine this hypothesis, we compared cellular aggregation capacities between WT and *csp* mutant strains of *L. monocytogenes* EGDe. To do this, stationary phase cultures that were statically incubated for 24 h were monitored for aggregation visually and through optical density measurement (Figure 5). Under these conditions the WT strain displayed complete aggregation whereas no significant aggregation was observed for the Δ*cspABD* (no *csp* genes), Δ*cspBD* (*cspA*), and Δ*cspAB* (*cspD*) strains. The Δ*cspAD* (*cspB*) strain although able to aggregate the levels of cellular aggregation it exhibited were significantly reduced compared to WT. Trends observed in aggregation phenotypes displayed by the different *csp* mutants interestingly also appear to correlate with their ActA levels as determined by Western blot analysis in BHI cultured bacteria of these mutants (Figure 3A). The Δ*cspABD* and Δ*cspAB* strains that produced no ActA as well as the Δ*cspBD* displaying the lowest detected ActA amounts were all unable to aggregate. In contrast the Δ*cspAD*, which produced the highest ActA amounts among the mutants was the only *csp* mutant capable of aggregation. Overall, these results indicated that Csps are also an important determinant of cellular aggregation in *L. monocytogenes* and this might involve Csp-dependent expression of cell surface proteins including ActA.

### Csp loss abolishes flagella production and swarming motility

**Figure 6:**
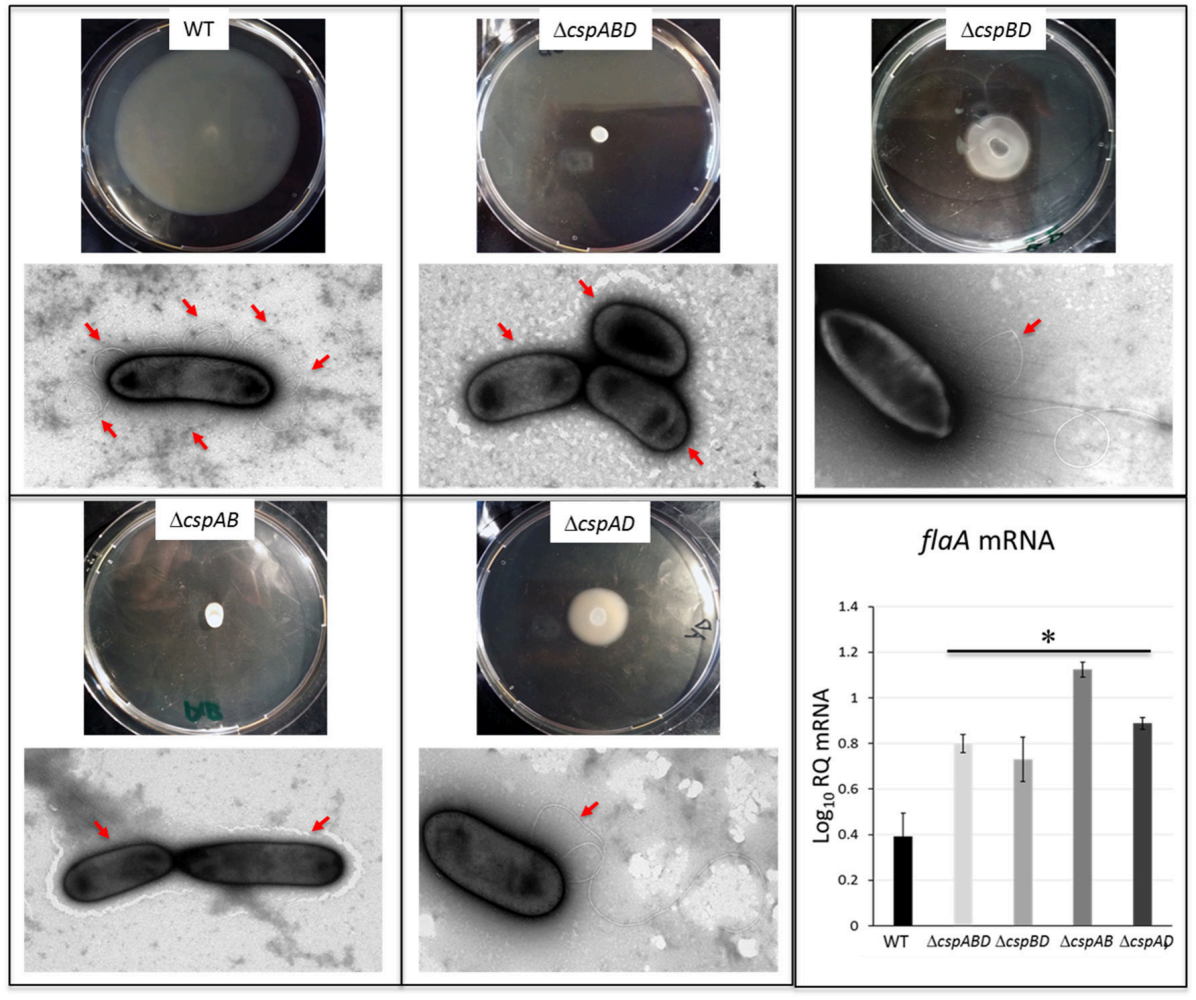
Impact of Csp loss on swarming motility, surface flagellation and *flaA* mRNA levels. Representative images of swarming motility assessment from bacteria cultures spotted on 0.25% BHI agar and incubated at 25°C for 48 h and transmission electron microscopy analysis for bacterium-associated flagella, as well as qRT-PCR quantification of *flaA* mRNA in EGDe WT and *csp* mutants cultivated to stationary phase in BHI at 25°C. Relative quantity (RQ) of *flaA* mRNA levels normalized 16S rRNA and expressed relative to an EGDe WT strain derived mRNA calibrator sample are shown. Data showing the means (bars) and standard deviations (error bars) of three independent biological experiment are presented. ^*^p < 0.05 based on one-way ANOVA and Tukey *post-hoc* test comparison relative to the WT strain. Presence or lack of flagella on cell surfaces is highlighted by red arrows.

The production of flagella and expression of motility contributes to virulence and cellular aggregation phenotypes in *L. monocytogenes*. Assessing for swarming motility and surface flagellation we also found striking differences between *csp* mutants and the WT strain of *L. monocytogenes* EGDe. Although the WT strain showed swarming motility at 25°C, the Δ*cspABD* (without Csps) and Δ*cspAB* (*cspD*) were not motile (Figure 6). The Δ*cspBD* (*cspA*) and Δ*cspAD* (*cspB*) strains although capable of swarming motility did so to visibly reduced extents when compared to the WT strain. Electron microscopic examination showed that while the motile WT strain exhibited peritrichous flagellation there were no flagella observed on the surfaces of the non-motile Δ*cspABD* and Δ*cspAB* mutants (Figure 6). In the weakly motile Δ*cspBD* and Δ*cspAD* strains flagellation was detected but at a low frequency compared to the WT strain. While almost all examined WT cells showed peritrichous flagellation, surface flagella expression could only be observed in 20–30% of all cells from these two *csp* mutants examined. Moreover, in those cells that were flagellated there was relatively fewer flagella per cell observed compared to the WT strain (Figure 6). To further explore the basis for these flagellation differences we also quantified *flaA* mRNA, which encodes flagellin, the main structural component for flagella, in the WT and *csp* mutant strains grown in BHI cultures grown at 25°C. Unexpectedly we found that *flaA* mRNA levels were significantly higher (*P* < 0.05) than those in the WT strain for all of the *csp* mutants including the Δ*cspABD* and Δ*cspAB* strains, which completely lack flagella (Figure 6). This observation indicated that although *flaA* mRNA was present in the *csp* mutants, it might be that it is not being efficiently translated to make the flagellin proteins. Such a scenario might lead to the reduced or lack of flagellation as observed in the *csp* mutants. Overall these observations therefore indicated that *csp* genes, particularly *cspA* and *cspB*, also influence flagella production and extracellular motility in *L. monocytogenes*.

## Discussion

Bacteria presumably use Csp proteins for the transcriptional and translational regulation of various genes to promote a wide range of physiological functions including stress resistance and virulence associated responses. To date Csps have been found important for pathogenicity in different bacteria including *Enterococcus fecalis, Staphylococcus aureus, Salmonella enterica, Brucella melitensis*, and *csp* gene deletions in such organisms were shown to cause attenuated virulence phenotypes (Michaux et al., 2012, 2017; Sahukhal and Elasri, 2014; Wang et al., 2016). On the other hand the phenotypic roles and molecular functional mechanisms associated with Csps in *L. monocytogenes* are not yet fully illuminated. In our present studies we show that Csps are not only involved in facilitating virulence but they also promote cellular aggregation as well as flagella-based motility, properties which are also important for survival of this bacterium outside the host environments.

We show here that a Δ*cspABD* mutation that removes all the *csp* genes also severely compromises pathogenicity of *L. monocytogenes* in human macrophages and zebra fish embryos. A Δ*cspABD* mutant shows a non-lethal phenotype when tested in a zebrafish embryo based infection model, while the WT induces 100% mortality of zebra fish embryos infected under similar conditions. Moreover, such a mutant while displaying limited intracellular growth during 6 h of infection could not be detected after 24 h of infection of a human derived macrophage cell line. In contrast, the WT *L. monocytogenes* strain efficiently multiplies and persists over 24 h of infection of such a human macrophage cell line. The phenotypic involvement and level of contribution of each of the three *L. monocytogenes* Csps was also confirmed through analysis of double *csp* gene deletion mutants that express single rather than all the three *csp* genes. Overall there appears to be a hierarchical phenotypic trend observed ranging from maximal virulence in the WT strain with all three *csp* genes, to variable but reduced virulence level in the double *csp* gene deletion mutants expressing single Csps and complete virulence attenuation in the Δ*cspABD* deletion mutant lacking all the three *csp* genes. As previously shown all *csp* mutants examined have no growth defects compared to the WT strain under optimal laboratory conditions in BHI at 37°C (Loepfe et al., 2010), our observations here thus suggest that inside human macrophages without Csps, *L. monocytogenes* either: (i) has an impaired ability to survive and grow, (ii) has a reduced capacity to spread from cell to cell, or (iii) gets cleared at a higher rate from host cells.

Consistent with Csps being involved in *L. monocytogenes* virulence gene expression modulation we previously showed that the *csp* mutants examined here produce low amounts of the virulence protein LLO, and this is in part linked to reduced amounts and low stability of the *hly* mRNA encoding for the LLO proteins observed without Csps in this bacterium (Schärer et al., 2013). In addition such *L. monocytogenes csp* mutants are also rendered more susceptible to oxidative stress conditions by the lack of Csps (Loepfe et al., 2010). Our findings here now revealed that besides such defects, there are also extensive defects in production of other virulence factors including ActA among the *csp* mutants. Taken together without *csp* genes, *L. monocytogenes* might therefore have a reduced ability to proliferate within host cells due to an impaired capacity to survive and escape from the hostile environment of the phagocytic vacuole coupled with an inability to spread to neighboring cells due to attenuated ActA production. Meanwhile the fact that we could not detect the Δ*cspABD* mutant in infected THP-1 macrophages after 24 h of infection might mean that there is complete destruction of the mutant within infected macrophages due to its inability to spread to neighboring cells.

Phenotypes observed with mutant strains carrying individual *csp* genes did not only provide functional confirmation for all the three *csp* genes in promoting *L. monocytogenes* virulence but they also showed that individual Csps do not function equally in this respect. Our observation that none of the *csp* genes expressed individually can recapitulate WT strain virulence but they all restore the phenotypes to levels above a Δ*cspABD* mutant without Csps, indicates that maximal phenotypic expression of virulence requires the functional activities of all the three Csps in *L. monocytogenes*. Meanwhile with respect to the different *csp* genes there was a general phenotypic trend observed showing that *cspB* followed by *cspD* are the genes with largest functional contributions to virulence, whereas the *cspA* gene, which is important in cold growth seems to have the least functional contributions to virulence. Based on this our findings thus indicated that despite being highly conserved the three *L. monocytogenes* Csps are not functionally equivalent nor are they completely redundant. The variable contribution of Csps to *L. monocytogenes* virulence could be down to variable Csp expression, differences in Csp regulated gene targets, or nucleic acid binding efficiencies. Under optimal growth conditions in BHI at 37°C we have previously observed that *csp* genes are variably expressed with *cspB* and *cspD* transcripts showing 20- and 4-fold, respectively, higher abundance than *cspA* mRNA in *L. monocytogenes* EGDe (Schmid et al., 2009). Such differences in *csp* gene expression at 37°C a temperature applied in our studies might therefore be part of the explanation behind the virulence phenotypic trends observed for the different Csps in the current study.

In other microorganisms Csp-dependent increases in the mRNA levels of various genes are linked to Csps either promoting the transcription or mRNA stability of transcripts derived from their target genes (Jiang et al., 1997; Bae et al., 2000; Feng et al., 2001; Michaux et al., 2017). Apart from increasing transcript levels, Csps can further increase protein synthesis through direct interactions with transcripts leading to translation promotion either by facilitating translation initiation or through destabilization of translation inhibiting mRNA secondary structures (Jiang et al., 1997; Bae et al., 2000). Since we had observed that virulence and macrophage persistence and growth were defective in *csp* mutants, we also assessed the *csp* mutants for the expression patterns of some of the known *L. monocytogenes* virulence genes at mRNA and protein levels. Overall the expression patterns observed for PrfA, LLO, ActA, Mpl, and PlcA (PI-PLC activity) in the Δ*cspABD* mutant compared to the WT is suggestive of at least two different mechanisms of interaction between Csps and their targets. In the case of *prfA, hly, plcA*, and *mpl* and the corresponding proteins (PrfA, LLO, and Mpl) or their activity (PI-PLC) we observed decreased amounts of mRNA as well as protein levels in the Δ*cspABD* mutant compared to the WT. Such findings are consistent with our earlier study that found reduced LLO protein expression and hemolytic activity in the Δ*cspABD* mutant (Schärer et al., 2013). In addition, there were recent transcriptome studies revealing an extensive regulation of various genes including those involved in virulence by Csps at the transcription level in *B. melitensis* and *S. enterica* (Wang et al., 2016; Michaux et al., 2017). Our observations here suggest that Csp-dependent regulation with respect to virulence gene expression might involve both direct and indirect mechanisms. Direct roles could be mediated through Csps directly targeting the *prfA, hly, mpl*, and *plcA* and *actA* mRNAs at transcriptional and translational levels. Csp-dependent regulation of PrfA expression further suggests an indirect transcriptional regulation of virulence genes that are under PrfA control including the *hly, plcA, mpl*, and *actA* genes.

In addition, we also found that in Δ*cspABD* and Δ*cspAB* mutants there was a complete lack of ActA and surface flagella although such strains showed significantly higher *actA* and *flaA* mRNA amounts compared to the WT strain. This expression pattern seems suggestive of a broken positive feedback loop where the lack of ActA and flagella might be sensed and the cells respond by increasing their expression at the transcript level, but these transcripts are not efficiently translated to proteins. Among the single Csp producing strains we similarly also found that while the CspB harboring Δ*cspAD* mutant had significantly higher *prfA, hly*, and *mpl* mRNA amounts, it exhibited similar or even lower amounts of their corresponding proteins when compared to the Δ*cspAD* mutant harboring CspD. Taken together these observations suggest possible involvement of the different Csps in post-transcriptional level expression regulation of these genes although such activities will need to be confirmed through further experimental work for the *L. monocytogenes* Csps. Meanwhile *L. monocytogenes* also regulates many of its virulence genes by mechanisms that involve the 5′-UTR of mRNA, including a thermoswitch in the *prfA* mRNA that prevents translation at low temperatures as well as other factors acting at post-transcription and translation levels (Johansson et al., 2002; Netterling et al., 2015; Reniere et al., 2016; Lebreton and Cossart, 2017). At this stage, it is tempting to speculate that Csps might be interacting with the *actA* and *flaA* mRNA in ways that promote their translation in *L. monocytogenes*. Possible but not yet proven mechanisms could also involve a role of Csps in the unmasking of the ribosomal binding site through mRNA structural destabilization or, in the case of *flaA*, Csps might somehow influence the interaction of the *flaA* transcript with an antisense mRNA transcribed from the P1 *mogR* promoter (Toledo-Arana et al., 2009).

Our findings here are also similar with reports from other bacteria linking Csp function to the regulation of flagella expression. Derman et al. previously reported a reduction in flagella formation and motility in *cspA* and *cspC* deleted mutants of *Clostridium botulinum* ATCC 3502 (Derman et al., 2015). A *csp* deletion mutant of *B. melitensis* showed increased *flaA* mRNA levels based on RNAseq (Wang et al., 2016), whilst an *Escherichia coli cspABGE* deletion mutant had reduced transcripts of the flagellar genes *flg* and *fli* based on a microarray analysis (Phadtare and Inouye, 2004). In external and food-associated environments flagella expression regulation through Csps and other gene expression regulators is also important for cold growth and biofilm production. The expression of Csps and flagella in *L. monocytogenes* gets induced at low temperature and these responses have been found important for cold growth, whilst flagella have also been shown to influence surface attachment and biofilm formation in this bacterium (Vatanyoopaisarn et al., 2000; Lemon et al., 2007; Schmid et al., 2009; Mattila et al., 2011). Although Csp involvement in biofilm production has not yet been reported in *L. monocytogenes*, Csps have been found important for biofilm production in bacteria such as *S. enterica, S. aureus* and *Vibrio cholera* (Sahukhal and Elasri, 2014; Townsley et al., 2016; Michaux et al., 2017).

In another interesting observation we also found that ActA protein expression patterns as determined by Western blot were reflected in the cell aggregation patterns observed in different *L. monocytogenes csp* mutants. In Δ*cspABD* and Δ*cspAB* lacking ActA as well as the Δ*cspBD* mutant containing the lowest amounts of ActA proteins we observed almost no bacterial aggregation. In contrast the Δ*cspAD* and WT strains showing highest amounts of ActA showed bacterial aggregation. Besides promoting intracellular motility and cell to cell dissemination, the surface protein ActA also mediates cellular aggregation and has been found to promote biofilm formation in *L. monocytogenes* (Travier et al., 2013). It would thus appear that similar to what was previously observed for PrfA, Csps also indirectly contribute to ActA-dependent aggregation in *L. monocytogenes* (Travier et al., 2013). Apart from altered ActA expression, the impaired aggregation phenotypes exhibited by the *L. monocytogenes csp* mutants could also have been due to the altered surface flagellation patterns exhibited among the different *csp* mutants, which either completely lacked (Δ*cspABD* and Δ*cspAB*) or showed reduced (Δ*cspBD* and Δ*cspAD*) surface flagellation. Cell surface flagella might contribute to cellular aggregation processes based on their roles in mediating surface attachment of *L. monocytogenes* cells (Vatanyoopaisarn et al., 2000; Lemon et al., 2007).

In conclusion our studies have shown that Csp functions are important for virulence as well as cell aggregation and flagella-based motility of *L. monocytogenes*. Our study shows for the first time in *L. monocytogenes* that Csp functions are involved in facilitating aggregation, flagella biosynthesis, and consequently extracellular motility. Notably the three Csps of *L. monocytogenes*, although highly conserved, clearly show both redundancy and variable functional relevance in enabling the Csp associated phenotypes. CspA has been found to be most important in cold adaptation and CspD in osmotic stress adaptation (Schmid et al., 2009), while this study suggests that CspB is the most relevant Csp with regard to virulence, aggregation and motility in *L. monocytogenes*. In conclusion, whilst the exact mechanisms through which Csps are involved in gene expression regulation in this bacterium remain to be investigated, we currently hypothesize based on molecular functions assigned to Csps from other bacteria, that the phenotypic roles associated with Csps in *L. monocytogenes* are most likely also linked to the involvement of these proteins in regulation of gene expression at both transcriptional and post-transcriptional levels in this bacterium.

## Author contributions

TT designed and supervised the study. AE performed the experiments. TT, AE, CG, and AO analyzed the data and wrote the manuscript.

### Conflict of interest statement

The authors declare that the research was conducted in the absence of any commercial or financial relationships that could be construed as a potential conflict of interest.

## References

[B1] Allerberger F. Wagner M. (2010). Listeriosis: a resurgent foodborne infection. Clin. Microbiol. Infect. 16, 16–23. 10.1111/j.1469-0691.2009.03109.x 20002687

[B2] Anonymous (2016). The European Union summary report on trends and sources of zoonoses, zoonotic agents and food-borne outbreaks in 2015. EFSA J. 14:4634 10.2903/j.efsa.2015.4329 PMC7009962 32625371

[B3] Bae W. Xia B. Inouye M. Severinov K. (2000). *Escherichia coli* CspA-family RNA chaperones are transcription antiterminators. Proc. Natl. Acad. Sci. U.S.A. 97, 7784–7789. 10.1073/pnas.97.14.7784 10884409 PMC16622

[B4] Batte J. L. Samanta D. Elasri M. O. (2016). MsaB activates capsule production at the transcription level in *Staphylococcus aureus*. Microbiology 162, 575–589. 10.1099/mic.0.000243 26781313 PMC4891993

[B5] Bigot A. Pagniez H. Botton E. Frehel C. Dubail I. Jacquet C.. (2005). Role of FliF and FliI of *Listeria monocytogenes* in flagellar assembly and pathogenicity. Infect. Immun. 73, 5530–5539. 10.1128/IAI.73.9.5530-5539.2005 16113269 PMC1231047

[B6] Camilli A. Tilney L. G. Portnoy D. A. (1993). Dual roles of plcA in *Listeria monocytogenes* pathogenesis. Mol. Microbiol. 8, 143–157. 10.1111/j.1365-2958.1993.tb01211.x 8388529 PMC4836944

[B7] Chan Y. C. Raengpradub S. Boor K. J. Wiedmann M. (2007). Microarray-based characterization of the *Listeria monocytogenes* cold regulon in log- and stationary-phase cells. Appl. Environ. Microbiol. 73, 6484–6498. 10.1128/AEM.00897-07 17720827 PMC2075049

[B8] de las Heras A. Cain R. J. Bielecka M. K. Vazquez-Boland J. A. (2011). Regulation of Listeria virulence: PrfA master and commander. Curr. Opin. Microbiol. 14, 118–127. 10.1016/j.mib.2011.01.005 21388862

[B9] Derman Y. Söderholm H. Lindström M. Korkeala H. (2015). Role of *csp* genes in NaCl, pH, and ethanol stress response and motility in *Clostridium botulinum* ATCC 3502. Food Microbiol. 46, 463–470. 10.1016/j.fm.2014.09.004 25475316

[B10] Dons L. Eriksson E. Jin Y. Rottenberg M. E. Kristensson K. Larsen C. N.. (2004). Role of flagellin and the two-component CheA/CheY system of *Listeria monocytogenes* in host cell invasion and virulence. Infect. Immun. 72, 3237–3244. 10.1128/IAI.72.6.3237-3244.2004 15155625 PMC415653

[B11] Dramsi S. Biswas I. Maguin E. Braun L. Mastroeni P. Cossart P. (1995). Entry of *Listeria monocytogenes* into hepatocytes requires expression of inIB, a surface protein of the internalin multigene family. Mol. Microbiol. 16, 251–261. 10.1111/j.1365-2958.1995.tb02297.x 7565087

[B12] Duan Q. Zhou M. Zhu L. Zhu G. (2013). Flagella and bacterial pathogenicity. J. Basic Microbiol. 53, 1–8. 10.1002/jobm.201100335 22359233

[B13] Dussurget O. (2008). New insights into determinants of *Listeria monocytogenes* virulence. Int. Rev. Cell Mol. Biol. 270, 1–38. 10.1016/S1937-6448(08)01401-9 19081533

[B14] Feng Y. Huang H. Liao J. Cohen S. N. (2001). *Escherichia coli* poly(A)-binding proteins that interact with components of degradosomes or impede RNA decay mediated by polynucleotide phosphorylase and RNase, E. J. Biol. Chem. 276, 31651–31656. 10.1074/jbc.M102855200 11390393

[B15] Freitag N. E. Port G. C. Miner M. D. (2009). *Listeria monocytogenes*- from saprophyte to intracellular pathogen. Nat. Rev. Microbiol. 7, 623–628. 10.1038/nrmicro2171 19648949 PMC2813567

[B16] Gaillard J. L. Berche P. Frehel C. Gouin E. Cossart P. (1991). Entry of L. monocytogenes into cells is mediated by internalin, a repeat protein reminiscent of surface antigens from gram-positive cocci. Cell 65, 1127–1141. 10.1016/0092-8674(91)90009-N 1905979

[B17] Glaser P. Frangeul L. Buchrieser C. Rusniok C. Amend A. Baquero F.. (2001). Comparative genomics of Listeria species. Science 294, 849–852. 10.1126/science.1063447 11679669

[B18] Horn G. Hofweber R. Kremer W. Kalbitzer H. R. (2007). Structure and function of bacterial cold shock proteins. Cell. Mol. Life Sci. 64, 1457–1470. 10.1007/s00018-007-6388-4 17437059 PMC11138454

[B19] Jami M. G. M. Zunabovic M. Domig K. J. (2015). *Listeria monocytogenes* in aquatic food products—a review. Comp. Rev. Food Sci. Food Saf. 13, 798–813. 10.1111/1541-4337.12092

[B20] Jiang W. Hou Y. Inouye M. (1997). CspA, the major cold-shock protein of *Escherichia coli*, is an RNA chaperone. J. Biol. Chem. 272, 196–202. 10.1074/jbc.272.1.196 8995247

[B21] Johansson J. Mandin P. Renzoni A. Chiaruttini C. Springer M. Cossart P. (2002). An RNA thermosensor controls expression of virulence genes in *Listeria monocytogenes*. Cell 110, 551–561. 10.1016/S0092-8674(02)00905-4 12230973

[B22] Josenhans C. Suerbaum S. (2002). The role of motility as a virulence factor in bacteria. Int. J. Med. Microbiol. 291, 605–614. 10.1078/1438-4221-00173 12008914

[B23] Keto-Timonen R. Hietala N. Palonen E. Hakakorpi A. Lindstrom M. Korkeala H. (2016). Cold shock proteins: a minireview with special emphasis on Csp-family of Enteropathogenic *yersinia*. Front. Microbiol. 7:1151. 10.3389/fmicb.2016.01151 27499753 PMC4956666

[B24] Kimmel C. B. Ballard W. W. Kimmel S. R. Ullmann B. Schilling T. F. (1995). Stages of embryonic development of the zebrafish. Dev. Dyn. 203, 253–310. 10.1002/aja.1002030302 8589427

[B25] Kocks C. Gouin E. Tabouret M. Berche P. Ohayon H. Cossart P. (1992). *L. monocytogenes*-induced actin assembly requires the actA gene product, a surface protein. Cell 68, 521–531. 10.1016/0092-8674(92)90188-I 1739966

[B26] Kramer M. N. Coto D. Weidner J. D. (2005). The science of recalls. Meat Sci. 71, 158–163. 10.1016/j.meatsci.2005.04.001 22064061

[B27] Lauer P. Chow M. Y. Loessner M. J. Portnoy D. A. Calendar R. (2002). Construction, characterization, and use of two *Listeria monocytogenes* site-specific phage integration vectors. J. Bacteriol. 184, 4177–4186. 10.1128/JB.184.15.4177-4186.2002 12107135 PMC135211

[B28] Lebreton A. Cossart P. (2017). RNA- and protein-mediated control of *Listeria monocytogenes* virulence gene expression. RNA Biol. 14, 460–470. 10.1080/15476286.2016.1189069 27217337 PMC5449094

[B29] Lemon K. P. Higgins D. E. Kolter R. (2007). Flagellar motility is critical for *Listeria monocytogenes* biofilm formation. J. Bacteriol. 189, 4418–4424. 10.1128/JB.01967-06 17416647 PMC1913361

[B30] Levraud J. P. Disson O. Kissa K. Bonne I. Cossart P. Herbomel P.. (2009). Real-time observation of *Listeria monocytogenes*-phagocyte interactions in living zebrafish larvae. Infect. Immun. 77, 3651–3660. 10.1128/IAI.00408-09 19546195 PMC2738018

[B31] Loepfe C. Raimann E. Stephan R. Tasara T. (2010). Reduced host cell invasiveness and oxidative stress tolerance in double and triple csp gene family deletion mutants of *Listeria monocytogenes*. Foodborne Pathog. Dis. 7, 775–783. 10.1089/fpd.2009.0458 20184451

[B32] Mattila M. Lindstrom M. Somervuo P. Markkula A. Korkeala H. (2011). Role of flhA and motA in growth of *Listeria monocytogenes* at low temperatures. Int. J. Food Microbiol. 148, 177–183. 10.1016/j.ijfoodmicro.2011.05.022 21683466

[B33] Melo J. Andrew P. W. Faleiro M. L. (2015). *Listeria monocytogenes* in cheese and the dairy environment remains a food safety challenge: the role of stress responses. Food Res. Internat. 67, 75–90. 10.1016/j.foodres.2014.10.031

[B34] Michaux C. Holmqvist E. Vasicek E. Sharan M. Barquist L. Westermann A. J.. (2017). RNA target profiles direct the discovery of virulence functions for the cold-shock proteins CspC and CspE. Proc. Natl. Acad. Sci. U.S.A. 114, 6824–6829. 10.1073/pnas.1620772114 28611217 PMC5495234

[B35] Michaux C. Martini C. Shioya K. Ahmed Lecheheb S. Budin-Verneuil A. Cosette P.. (2012). CspR, a cold shock RNA-binding protein involved in the long-term survival and the virulence of *Enterococcus faecalis*. J. Bacteriol. 194, 6900–6908. 10.1128/JB.01673-12 23086208 PMC3510560

[B36] Netterling S. Bareclev C. Vaitkevicius K. Johansson J. (2015). RNA helicase important for *Listeria monocytogenes* hemolytic activity and virulence factor expression. Infect. Immun. 84, 67–76. 10.1128/IAI.00849-15 26483402 PMC4693997

[B37] O'Neil H. S. Marquis H. (2006). *Listeria monocytogenes* flagella are used for motility, not as adhesins, to increase host cell invasion. Infect. Immun. 74, 6675–6681. 10.1128/IAI.00886-06 16982842 PMC1698079

[B38] Phadtare S. Inouye M. (2004). Genome-wide transcriptional analysis of the cold shock response in wild-type and cold-sensitive, quadruple-csp-deletion strains of *Escherichia coli*. J. Bacteriol. 186, 7007–7014. 10.1128/JB.186.20.7007-7014.2004 15466053 PMC522181

[B39] Phadtare S. Severinov K. (2010). RNA remodeling and gene regulation by cold shock proteins. RNA Biol. 7, 788–795. 10.4161/rna.7.6.13482 21045540 PMC3073336

[B40] Portnoy D. A. Jacks P. S. Hinrichs D. J. (1988). Role of hemolysin for the intracellular growth of Listeria monocytogenes. J. Exp. Med. 167, 1459–1471. 10.1084/jem.167.4.1459 2833557 PMC2188911

[B41] Prajsnar T. K. Cunliffe V. T. Foster S. J. Renshaw S. A. (2008). A novel vertebrate model of *Staphylococcus aureus* infection reveals phagocyte-dependent resistance of zebrafish to non-host specialized pathogens. Cell. Microbiol. 10, 2312–2325. 10.1111/j.1462-5822.2008.01213.x 18715285

[B42] Reniere M. L. Whiteley A. T. Portnoy D. A. (2016). An *in vivo* selection identifies *Listeria monocytogenes* genes required to sense the intracellular environment and activate virulence factor expression. PLoS Pathog. 12:e1005741. 10.1371/journal.ppat.1005741 27414028 PMC4945081

[B43] Sahukhal G. S. Elasri M. O. (2014). Identification and characterization of an operon, msaABCR, that controls virulence and biofilmdevelopment in *Staphylococcus aureus*. BMC Microbiol. 14:154. 10.1186/1471-2180-14-154 24915884 PMC4229872

[B44] Schärer K. Stephan R. Tasara T. (2013). Cold shock proteins contribute to the regulation of listeriolysin O production in *Listeria monocytogenes*. Foodborne Pathog. Dis. 10, 1023–1029. 10.1089/fpd.2013.1562 23952475

[B45] Schmid B. Klumpp J. Raimann E. Loessner M. J. Stephan R. Tasara T. (2009). Role of cold shock proteins in growth of *Listeria monocytogenes* under cold and osmotic stress conditions. Appl. Environ. Microbiol. 75, 1621–1627. 10.1128/AEM.02154-08 19151183 PMC2655451

[B46] Shen A. Higgins D. E. (2005). The 5′ untranslated region-mediated enhancement of intracellular listeriolysin O production is required for *Listeria monocytogenes* pathogenicity. Mol. Microbiol. 57, 1460–1473. 10.1111/j.1365-2958.2005.04780.x 16102013

[B47] Silk B. J. Date K. A. Jackson K. A. Pouillot R. Holt K. G. Graves L. M.. (2012). Invasive listeriosis in the foodborne diseases active surveillance network (FoodNet), 2004-2009: further targeted prevention needed for higher-risk groups. Clin. Infect. Dis. 54(Suppl. 5), S396–S404. 10.1093/cid/cis268 22572660

[B48] Slepkov E. R. Pavinski Bitar A. Marquis H. (2010). Differentiation of propeptide residues regulating the compartmentalization, maturation and activity of the broad-range phospholipase C of *Listeria monocytogenes*. Biochem. J. 432, 557–563. 10.1042/BJ20100557 20879990 PMC3469326

[B49] Soni K. A. Nannapaneni R. Tasara T. (2011). The contribution of transcriptomic and proteomic analysis in elucidating stress adaptation responses of *Listeria monocytogenes*. Foodborne Pathog. Dis. 8, 843–852. 10.1089/fpd.2010.0746 21495855

[B50] Toledo-Arana A. Dussurget O. Nikitas G. Sesto N. Guet-Revillet H. Balestrino D.. (2009). The *Listeria* transcriptional landscape from saprophytism to virulence. Nature 459, 950–956. 10.1038/nature08080 19448609

[B51] Townsley L. Sison Mangus M. P. Mehic S. Yildiz F. H. (2016). Response of vibrio cholerae to low-temperature shifts: CspV regulation of type VI secretion, biofilm formation, and association with Zooplankton. Appl. Environ. Microbiol. 82, 4441–4452. 10.1128/AEM.00807-16 27208110 PMC4959209

[B52] Travier L. Guadagnini S. Gouin E. Dufour A. Chenal-Francisque V. Cossart P.. (2013). ActA promotes *Listeria monocytogenes* aggregation, intestinal colonization and carriage. PLoS Pathog. 9:e1003131. 10.1371/journal.ppat.1003131 23382675 PMC3561219

[B53] Vatanyoopaisarn S. Nazli A. Dodd C. E. Rees C. E. Waites W. M. (2000). Effect of flagella on initial attachment of *Listeria monocytogenes* to stainless steel. Appl. Environ. Microbiol. 66, 860–863. 10.1128/AEM.66.2.860-863.2000 10653766 PMC91911

[B54] Wang Z. Liu W. Wu T. Bie P. Wu Q. (2016). RNA-seq reveals the critical role of CspA in regulating *Brucella melitensis* metabolism and virulence. Sci. China Life Sci. 59, 417–424. 10.1007/s11427-015-4981-6 26740105

[B55] Wemekamp-Kamphuis H. H. Karatzas A. K. Wouters J. A. Abee T. (2002). Enhanced levels of cold shock proteins in *Listeria monocytogenes* LO28 upon exposure to low temperature and high hydrostatic pressure. Appl. Environ. Microbiol. 68, 456–463. 10.1128/AEM.68.2.456-463.2002 11823178 PMC126669

[B56] Widziolek M. Prajsnar T. K. Tazzyman S. Stafford G. P. Potempa J. Murdoch C. (2016). Zebrafish as a new model to study effects of periodontal pathogens on cardiovascular diseases. Sci. Rep. 6:36023. 10.1038/srep36023 27777406 PMC5078774

[B57] Yamanaka K. Zheng W. Crooke E. Wang Y. H. Inouye M. (2001). CspD, a novel DNA replication inhibitor induced during the stationary phase in *Escherichia coli*. Mol. Microbiol. 39, 1572–1584. 10.1046/j.1365-2958.2001.02345.x 11260474

