# Supplementary material for: Cold-Shock Domain Family Proteins (Csps) Are Involved in Regulation of Virulence, Cellular Aggregation, and Flagella-Based Motility in Listeria monocytogenes
Source: Front Cell Infect Microbiol. 2017 Oct 26;7:453. doi: 10.3389/fcimb.2017.00453 (PMC5662587; doi:10.3389/fcimb.2017.00453)
Supplement: Supplementary file 1 [file Image1.pdf]

Figure S1: Bar charts showing percentage uptake/invasion of *L. monocytogenes* EGDe WT and csp mutants into THP-1 at time point T0. The percentages are expressed relative to the original bacteria inoculum.

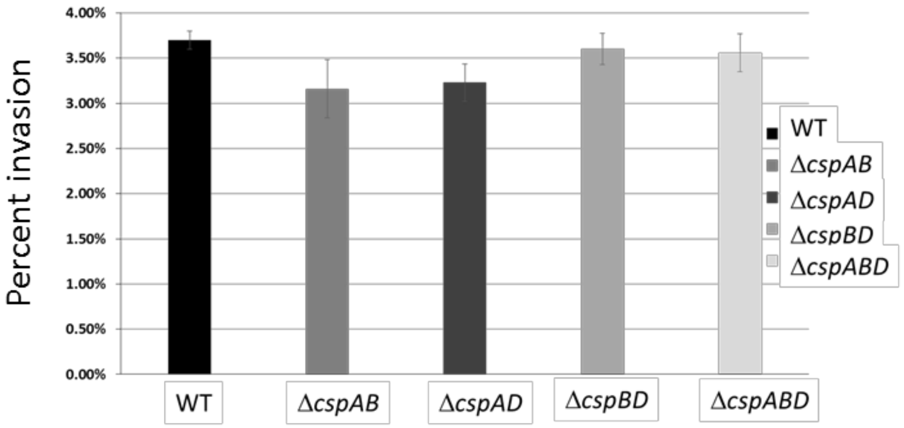

Figure S2: Representative uncropped images of Western blot analysis showing LLO and Mpl detection

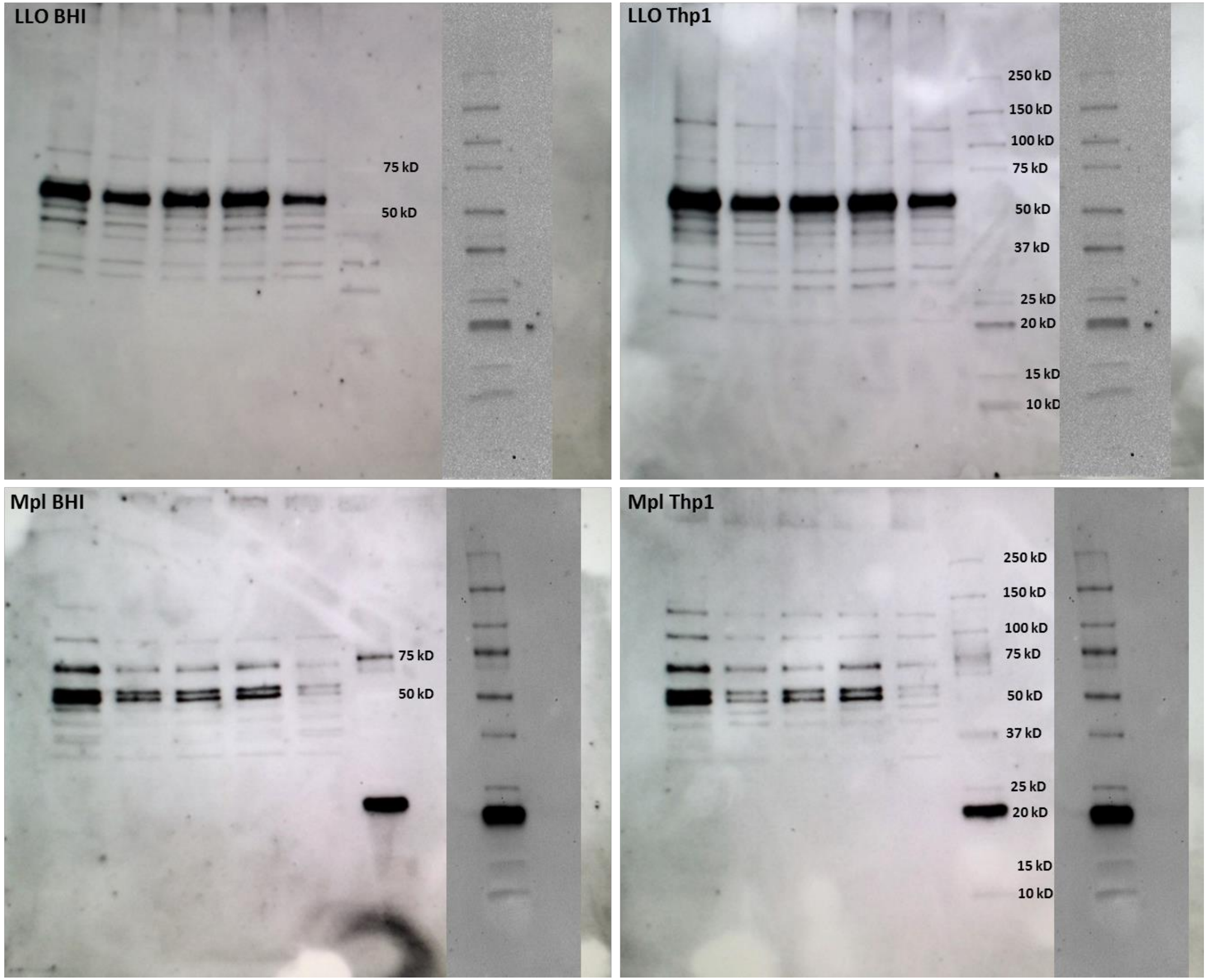

Figure S3: Representative uncropped images of Western blots showing PrfA and ActA detection

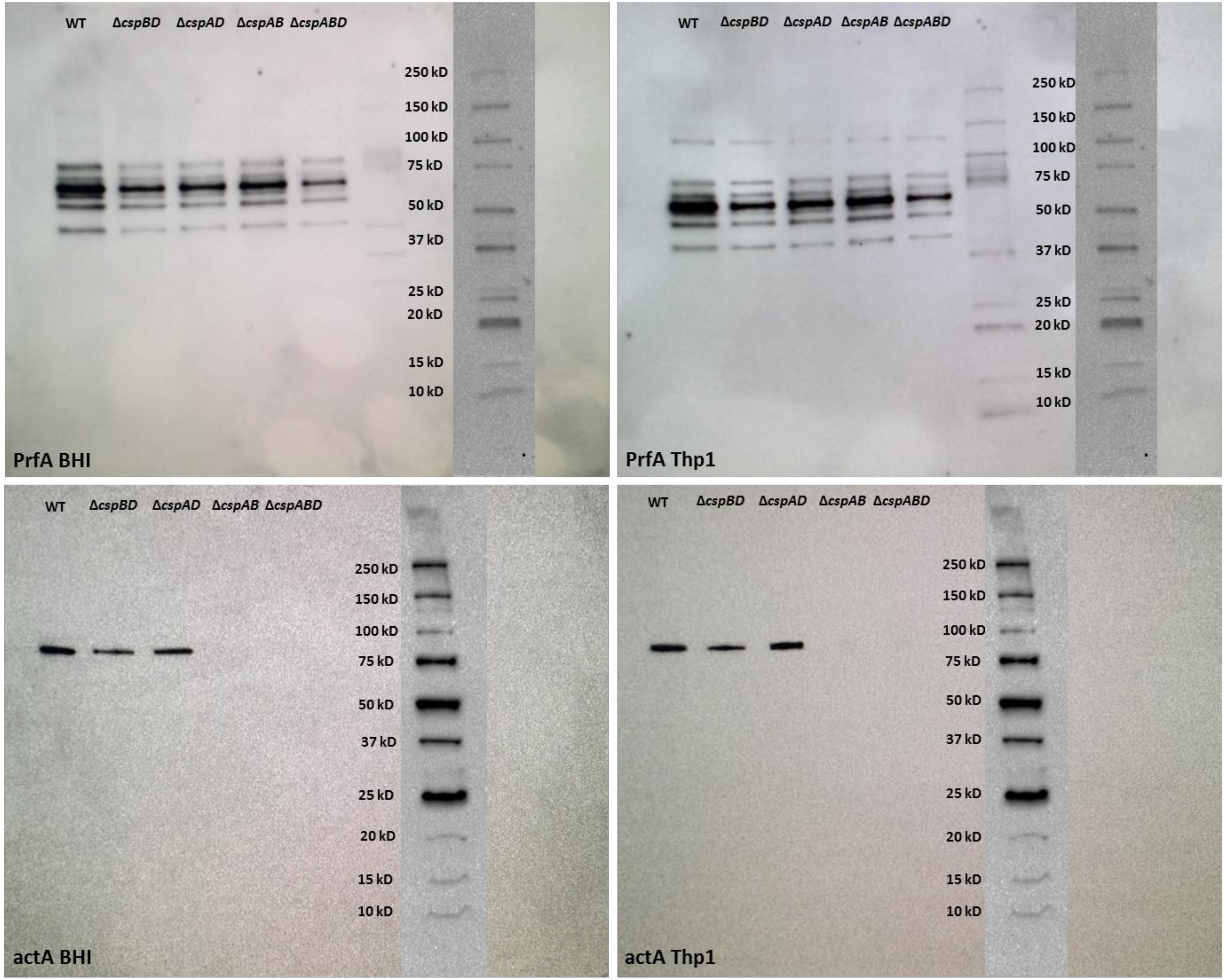

Figure S4: Representative uncropped images of Western blots showing P60 detection

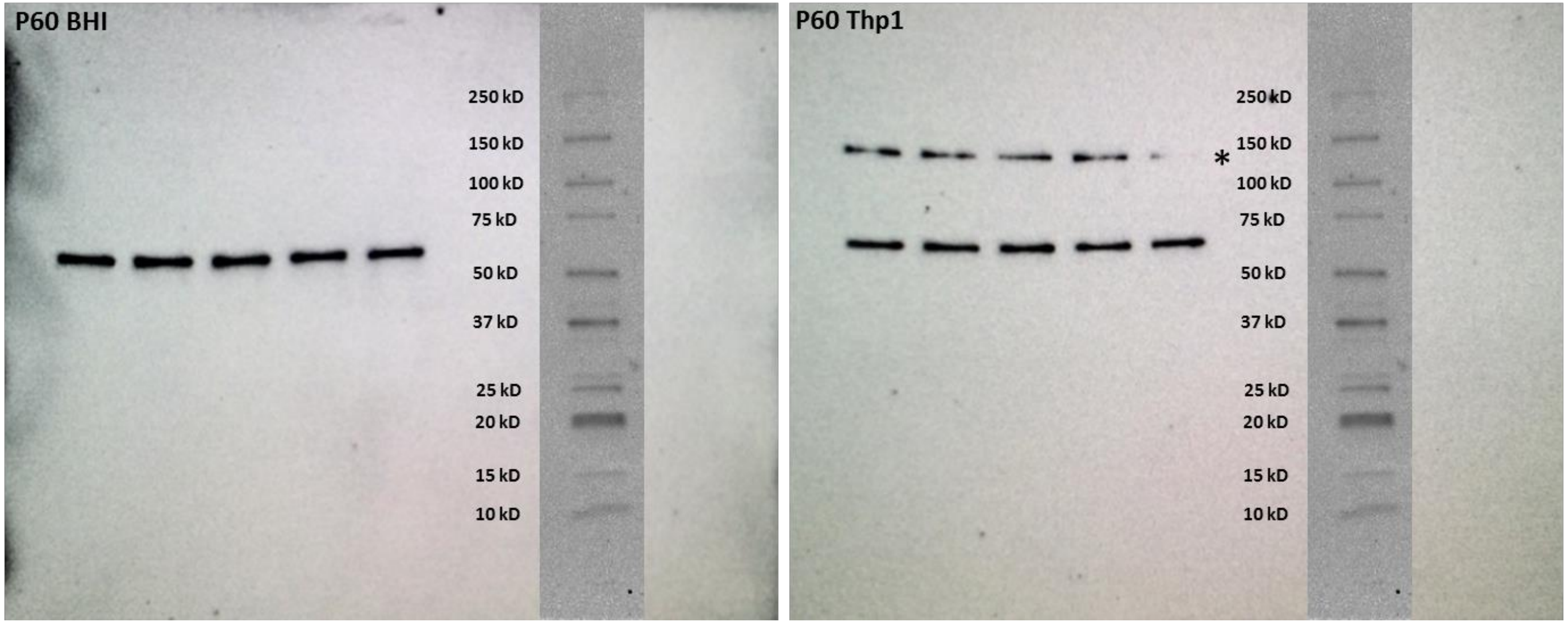

*Protein from Eukaryotic cells that cross reacts with the anti-P60 antibodyfrom
